# Chemical Kinetic and Mechanistic Investigation of Cobalt and Manganese Recovery from Aqueous Solutions by Ozone Oxidative Precipitation

**DOI:** 10.1021/acs.est.5c18743

**Published:** 2026-06-03

**Authors:** Younes Shekarian, Mohammad Rezaee, Sarma V. Pisupati

**Affiliations:** John and Willie Leone Family Department of Energy and Mineral Engineering, Center for Critical Minerals, College of Earth and Mineral Science, 8082 The Pennsylvania State University, University Park, Pennsylvania 16802, United States

**Keywords:** critical minerals; oxidative precipitation, coprecipitation, ozone, kinetic study

## Abstract

Cobalt (Co) and manganese (Mn) are designated as critical elements by the U.S. Department of the Interior, yet the mechanisms governing their recovery from aqueous systems remain poorly understood. Ozone-induced oxidative precipitation offers a promising, chemical-minimizing approach for recovering these elements from aqueous solutions. Building on our previously published, patent-pending, work, this study investigated the effect of gas flow rate, stirring rate, ion concentration, and temperature on Co–Mn precipitation performance using ozone. Model solutions replicating typical Co and Mn concentrations in aqueous secondary sources were prepared for controlled laboratory experiments. The influence of individual parameters was first evaluated, followed by a statistically designed parametric study. Results showed maximum Co recovery at a medium gas flow rate (1100 cc/min) and moderate temperature (50 °C), whereas Mn recovery was optimized at an elevated temperature (80 °C) and a high flow rate (2000 cc/min). Ozone mass-transfer limitations were described using the two-film theory, and measured volumetric mass transfer coefficient (K_L_a) values were consistent with the observed precipitation trends. Quantitatively, K_L_a increased from 0.0032 s^–1^ at 20 °C to 0.0057 s^–1^ at 40 °C, then decreased at higher temperatures due to reduced ozone solubility and stability. Similarly, increasing gas flow from 200 cc/min to 1400 cc/min raised K_L_a to 0.0147 s^–1^, with a slight decline at 2000 cc/min caused by incomplete ozone generation and accelerated decomposition. Kinetic modeling during the first 30 s identified that the pseudohomogeneous model best described the system, highlighting both ozone mass transfer and oxidant availability in solution as rate-limiting factors. Activation energies were calculated: 8.6 kJ/mol (Co) and −15.3 kJ/mol (Mn) in pure solutions, and 11.4 kJ/mol (Co) and −12.3 kJ/mol (Mn) in mixed solution, indicating a diffusion-controlled process. Notably, Co recovery in the mixed solution increased with temperature as Mn oxides (MnO_2_, MnOOH) formed nucleation sites that promoted Co adsorption and coprecipitation, a mechanism supported by SEM–EDS, XRD, FT–IR, XPS, and Zeta Potential analyses confirming the formation of Mn oxide phases facilitating surface-mediated Co capture. Overall, these findings provide mechanistic and kinetic insight into ozone-based Co–Mn recovery and establish a basis for process design and optimization in critical metal recovery applications.

## Graphical abstract

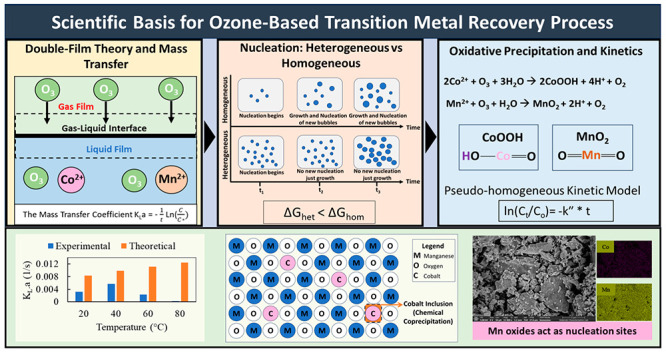

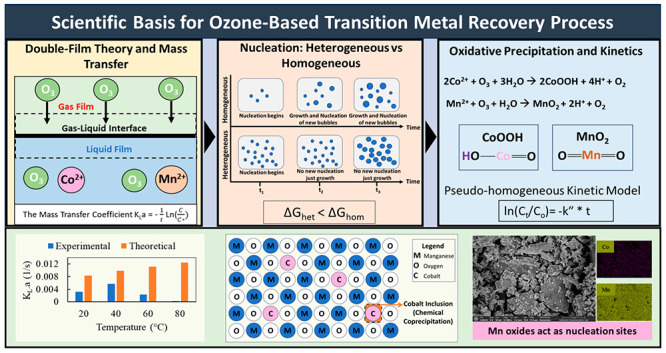

## 1 Introduction

Cobalt (Co) and manganese (Mn) are among the critical minerals listed by the U.S. Department of the Interior due to their essential role in modern technologies and the nation’s reliance on their foreign supplies.^1–3^ The demand for these transition metals is projected to grow significantly, largely driven by their applications in battery materials for electric vehicles.^4^ Domestic primary sources of these metals are limited, prompting increased interest in recovery from secondary sources. These include spent lithium-ion batteries, mixed hydroxide precipitates from nickel laterite processing, coal refuse, metallurgical byproducts and sludges, oil and gas produced water, acid mine drainage (AMD), and geothermal brine sources.^5–11^

The recovery of critical minerals from aqueous waste streams is essential for supporting the clean energy transition. Recent advancements in critical materials recovery have highlighted the need for innovative and sustainable approaches.^9–11^ Common techniques for recovering Co and Mn from aqueous solutions include chemical precipitation, adsorption, ion exchange, solvent extraction, electrochemical methods, and advanced oxidation processes such as ozonation, often combined in sequential or integrated flowsheets to improve selectivity and recovery.^9–23^ Staged precipitation is an effective approach for the selective recovery of elements, precipitating them in sequence and generating multiple valuable products.^24,25^ However, precipitation of Co and Mn from wastewater streams is challenging, as these elements do not precipitate under circumneutral pH. Their precipitation throughout the conventional hydroxide or ammoniacal treatment process starts at pH ∼ 9 and typically requires a pH of approximately 10.5 for high recovery.^26^ Alternatively, oxidative precipitation of Co and Mn has been investigated using a variety of oxidants, including hydrogen peroxide, SO_2_/O_2_ mixture, ozone, Caro’s acid, peroxydisulfuric acid, hypochlorite, and chlorite, each commonly used in separation processes for these metals.^10,12,15^ ^,^ ^18,27–31^ Building upon our previous work, to reduce chemical consumption, this study employs ozone oxidative precipitation to recover Co and Mn at circumneutral pH.^32^

Ozone (O_3_) is a powerful oxidizing agent with a high oxidation potential of 2.07 V (see Figure S1 and Table S1 in Supporting Information). In aqueous solution, dissolved ozone rapidly oxidizes Co^2+^ and Mn^2+^ to form insoluble oxyhydroxide or oxide phases, initiating precipitation; the detailed reaction pathways and precipitation mechanisms relevant to this process are summarized in Supporting Information (Section S1). Ozone has been widely applied in hydrometallurgical separations of Co and Mn from various resources. Several researchers have investigated the oxidative precipitation of Co and Mn using ozone. Ichlas et al.^33^ demonstrated oxidative precipitation of Co and Mn from mixed nickel–cobalt hydroxide precipitate (MHP) leach solutions, achieving complete Co–Mn removal at pH 5.0 with minimal Ni coprecipitation. Similarly, Sale et al.^34^ reported nearly complete Mn recovery (97–100%) from Li-ion battery leachates using ozone, avoiding secondary contaminants associated with other oxidants.^34^ In zinc hydrometallurgy, Jing et al.^13^ showed that iron oxidation in zinc leach solutions is controlled by ozone dissolution and pH.^16^ Tian et al.^27^ further demonstrated that Co oxidation is diffusion-controlled with rate enhancement at higher gas flow and stirring speeds, but largely unaffected by initial ion concentration and temperature.^27^ Oruê et al.^17^ and Cruz-Díaz et al.^21^ confirmed that Mn precipitation in acidic solution (pH < 2) follows a pseudohomogeneous mass-transfer model, with selectivity and rate governed by ozone availability and solution pH.^17,21^

Despite these advances, a significant knowledge gap remains in understanding how process parameters and their interactions influence the ozone-based oxidative precipitation of Co and Mn. Additionally, kinetic data specific to ozone-mediated Co and Mn precipitation remain scarce.^16–18,22^ Although previous studies have primarily examined ozone oxidation of single-metal systems (typically Co or Mn) in strongly acidic conditions, the mechanistic behavior and kinetic interactions of multimetal systems under near-neutral pH remain largely unexplored.

This work fills these gaps by simultaneously investigating Co and Mn oxidative precipitation using ozone in both pure and mixed solutions, representative of secondary aqueous resources such as AMD and industrial effluents. By integrating mass-transfer modeling (two-film theory), kinetic fitting (Linear, Higbie, and pseudohomogeneous models), and surface-mediated coprecipitation analysis, this study provides the first comprehensive assessment of the coupled chemical and physical mechanisms governing ozone-based recovery of Co–Mn under environmentally relevant conditions.

Accordingly, this study (i) investigates the effect of operational parameters (temperature, stirring rate, gas flow rate, and initial ion concentration) on Co–Mn precipitation performance using ozone; (ii) identifies the mechanism governing their precipitation and determines the kinetics of the process to obtain the scaleup parameters; and (iii) investigates whether Co and Mn precipitate independently or through coprecipitation and surface-mediated adsorption, using both solution chemistry analysis and experimental evidence. Together, these objectives provide a foundation for the design, optimization, and scale-up of ozone oxidative precipitation processes for Co–Mn recovery from complex aqueous systems.

## 2 Materials and Methods

### 2.1 Materials

Two separate stock solutions were prepared, one containing 10 ppm of Co and the other containing 50 ppm Mn, representing the concentration reported for AMD from sites in Pennsylvania.^1,28,35,36^ The pH of the stock solutions was adjusted to 7 to simulate our previous study in which the oxidative precipitation was performed on the neutralized AMD (i.e., pH 7) obtained from the two-staged carbonate precipitation process.^28^ In the staged precipitation, major competing constituents such as Fe, Al, and REEs, as well as suspended or biological materials, were removed prior to the ozone oxidative precipitation stage. This pretreatment minimizes competing ozone-consuming species, since organic compounds and suspended biological materials can consume ozone or ozone-derived radicals and may affect Co–Mn precipitation kinetics and precipitate purity. Therefore, the prepared pH 7 Co–Mn solutions were used to isolate the kinetics and mechanisms of Co–Mn recovery under conditions representative of the pretreated AMD stage. Following the oxidative precipitation experiments, precipitates were separated from the liquid phase via vacuum filtration using a 0.45 μm hydrophilic polyvinylidene fluoride (PVDF) filter (Durapore membrane - Millipore-Sigma). The collected precipitates were dried at a low temperature (70 °C) in a vacuum oven (−70 kPa) and stored sealed for subsequent characterization and analyses.

### 2.2 Methods

#### 2.2.1 Solution Chemistry

Ozone dissolution into the aqueous phase is governed by gas-liquid mass-transfer processes described by the two-film theory. As illustrated in Figure 1, ozone diffuses through boundary layers (or “films”) on both sides of the interface before chemically reacting (i.e., RS1-RS5 in Supporting Information) in the bulk liquid phase.^22,37,38^ In the gas phase, ozone concentration decreases from the bulk gas concentration (C_g_) to the interfacial gas concentration (C_gi_). At the interface, ozone transfers into the liquid phase and equilibrates with an interfacial dissolved concentration (C_li_), which is governed by Henry’s law. The ozone then diffuses through the liquid film to the bulk liquid concentration (C_l_). The driving force for mass transfer is the overall difference in concentration between the phases, typically expressed as the gradient (C*–C_l_), where C* is the equilibrium concentration in the liquid phase.^39,40^ This gradient determines the overall mass-transfer rate and the availability of ozone for oxidation and subsequent precipitation. Detailed theoretical (eq (S1−S3) in Supporting Information) and experimental (eq (S4−S5) in Supporting Information) calculations of the volumetric mass transfer coefficient (K_L_a) are provided in the Supporting Information (Section S1.3).

**Figure 1:**
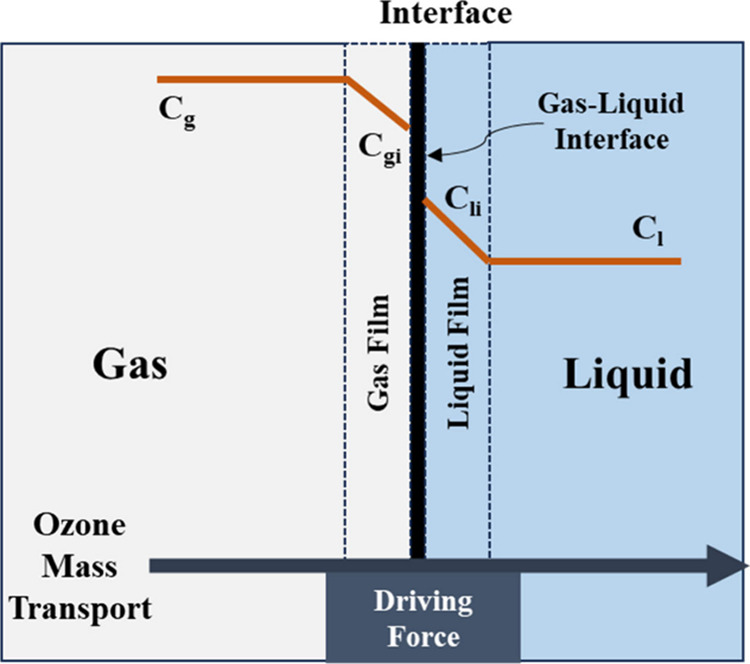
Schematic of ozone mass transfer across the gas–liquid interface (conceptually adopted from^39^). C_g_: bulk gas concentration; C_gi_: gas concentration at the gas–liquid interface; C_li_: liquid interfacial concentration; C_l_: bulk liquid concentration. The stepwise decrease in concentration represents the transport resistance in the gas and liquid films.

Once dissolved, ozone establishes the redox and speciation environment that governs metal precipitation. To assess the thermodynamic favorability of precipitation, the saturation index (SI) was calculated as

1 $$\mathrm{SI}=\log\left(\mathrm{IAP}/K_{\mathrm{sp}}\right)$$

where *IAP* is the Ion Activity Product, the product of the dissolved metal and oxide/hydroxide ions’ activities (or concentrations) in solution, and *K*_*sp*_ is the solubility product constant for the metal hydroxide or oxide at 25 °C. SI, speciation, and redox stability diagrams were generated using Visual MINTEQ and HSC Chemistry to evaluate the conditions favoring Co–Mn oxidative precipitation.

#### 2.2.2 Ozone Oxidative Precipitation

The effect of process parameters, including stirring rate, gas flow rate, initial ion concentration, and temperature, on oxidative precipitation of Co–Mn using ozone was systematically investigated. The stock solutions at pH 7 were prepared using ACS grade NaOH. Ozone wet-chemistry experiments were conducted using potassium iodide (KI), sodium thiosulfate (Na_2_S_2_O_3_), potassium periodate (KIO_3_), Sulfuric acid (H_2_SO_4_, 2 N), and starch indicator to measure the amount of residual ozone. For the oxidative ozone precipitation, two separate ozone generators (T-king Enaly Model, 1000 mg/h capacity) were used with 99% pure oxygen as the feed gas to purge ozone into Co and Mn solutions. The volumetric flow rate of ozone injected into the system was manually controlled by a flowmeter, directed into the solution using a porous ceramic diffuser in a 500 mL glass reactor. The O_2_/O_3_ gas mixture was continuously bubbled through the solution, and while redox potential (ORP) was continuously monitored throughout the experiments, the pH was maintained constant at the desired value. Samples were collected at multiple times: 0 s, 5 s, 15 s, 30 s, 45 s, 60 s, 90 s, 120 s, 5 min, 10 min, 15 min, 20 min, 30 min, 1 h, 2 h, 4 h, and 8 h. For each interval, 5 mL of the solution was sampled using a pipet and immediately filtered using EZFlow syringe filters with a 0.22 μm pore size. Following filtration, the filtrates were promptly acidified with 70% HNO_3_, to a final concentration of 5% (v/v) HNO_3_ to prevent further precipitation. During the oxidative precipitation process, ACS grade NaOH was used to maintain the solution pH at 7. The choice of process parameters and reagents for each stage was informed by our previous studies.^28,41^ The experimental setup is schematically illustrated in Figure 2.

**Figure 2:**
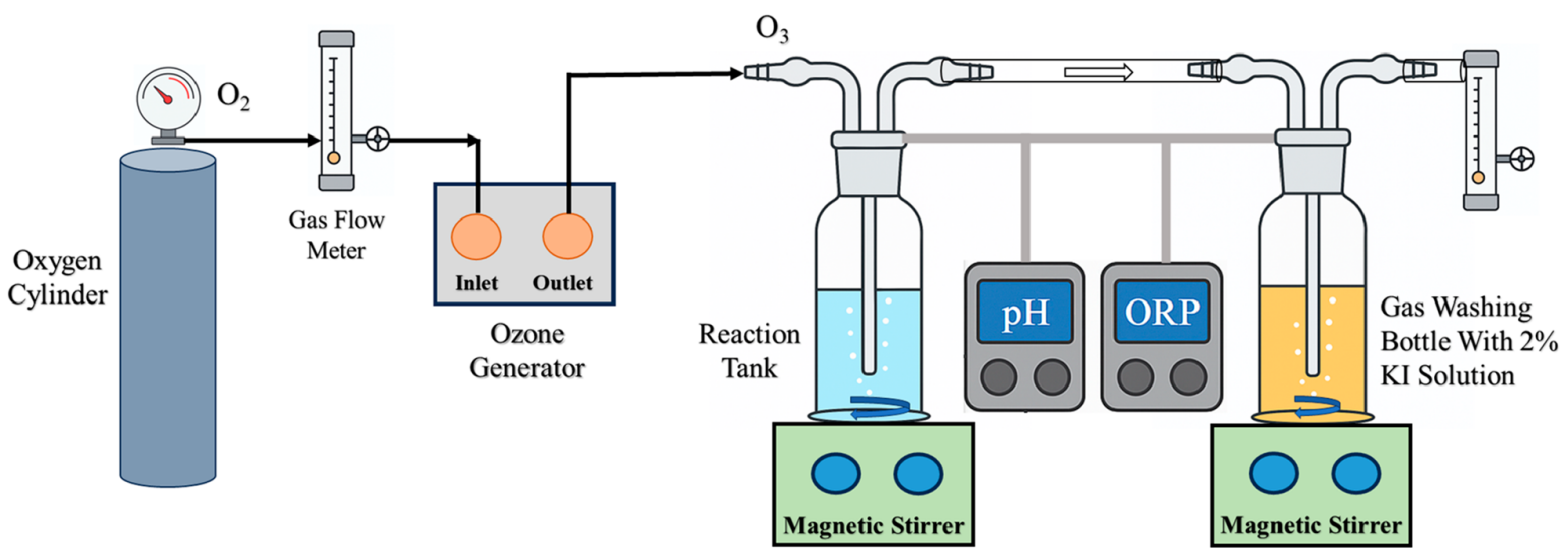
Schematic of the experimental setup for ozone oxidative precipitation.

#### 2.2.3 Kinetics of Precipitation

To study precipitation kinetics, a one-variable-at-a-time (OVAT) approach was employed. For each experiment, one parameter (flow rate, stirring rate, or ion concentration) varied while we kept the others constant. A total of 17 experiments were conducted for each element (Co and Mn) to study the effect of individual parameters. Various parameters studied in these experiments include flow rate (200, 800, 1400, and 2000 cc/min), stirring rate (0, 400, 800, 1200, and 1500 rpm), and initial Co–Mn concentration (5, 50, 500, and 5000 ppm). All experiments were performed in 500 mL solutions at room temperature (20 ± 2.5 °C). At selected time intervals, 5 mL samples were collected, filtered immediately, and acidified with HNO_3_ solution to hinder further precipitation. To evaluate the effect of temperature and determine the kinetic rate and activation energy (*E*_a_), kinetic experiments were conducted at four distinct temperatures (20, 40, 60, and 80 °C). In this study, three different kinetic models, including the Linear model, Higbie model, and Pseudohomogeneous, were used to calculate the rate and activation energy of precipitation reactions using ozone.^32^ While all three were considered for comparison, the pseudohomogeneous model provided the best fit to the experimental data and was therefore used as the primary model for interpreting reaction kinetics. Details of the pseudohomogeneous kinetic model (eq (S6)) and Arrhenius equation (eq (S7)) are given in the Supporting Information (Section S1.4).

#### 2.2.4 Process Optimization

To determine the effect of key process parameters and their interaction on the oxidative precipitation of Co–Mn, a statistically designed parametric study using the Box-Behnken method was performed on the prepared stock solutions. The experimental program evaluated the effects of the flow rate, stirring rate, and temperature. A total of 17 experiments (including 5 repeat tests at the center points) were performed for each element based on the 3-level experimental design (Table 1).

**Table 1:** Parameter Value Ranges Used for Box-Behnken Design

	Level		
Parameter	Low	Medium	High
Flow rate (cc/min)	200	1100	2000
Stirring rate (rpm)	0	400	800
Temperature (°C)	20	50	80

#### 2.2.5 Characterization and Data Analysis

Elemental content analysis of the filtrates collected at each test was conducted using an Agilent 7800 inductively coupled plasma mass spectrometer (ICP-MS) at the Penn State Center for Critical Minerals (C^2^ M). The analyses were performed in duplicate, using ICP-MS standard solutions (ICP-MS-68B-A-100, HPS) for quality control. Co–Mn precipitates were thoroughly characterized using X-ray diffraction (XRD), Fourier transform infrared (FTIR) spectroscopy, X-ray photoelectron spectroscopy (XPS), zeta potential (Malvern Zetasizer), and scanning electron microscopy with energy dispersive X-ray spectroscopy (SEM-EDS) at Penn State Material Characterization Laboratories (MCL).

The elemental recovery values were calculated based on the elemental concentrations and using Equation 2, where *C*_*i*_ is the concentration (mg/L) of the element of interest in the solution after filtration at each stage, and *C*_*f*_ is the concentration of the same element in the feed.

2 $$Recovery\left(\%\right)=100\cdot \left(1-\frac{C_{i}}{C_{f}}\right)$$

## 3 Results and Discussion

### 3.1 Solution Chemistry Study

Saturation index analysis was used to evaluate the thermodynamic favorability of Co–Mn oxidative precipitation under ozone treatment. As shown in Figure 3a, Co and Mn oxide phases reach positive SI values at low to near-neutral pH in the presence of ozone, whereas hydroxide precipitation requires elevated pH. The corresponding Eh–log­[M^2+^] diagram (Figure 3b) confirms that ozone provides sufficient redox potential for selective Co–Mn precipitation at pH 7. This was also supported by aqueous speciation diagrams (Figure S2) and Eh–pH (Pourbaix) diagrams (Figure S3) provided in the Supporting Information (Section S2.1).

**Figure 3:**
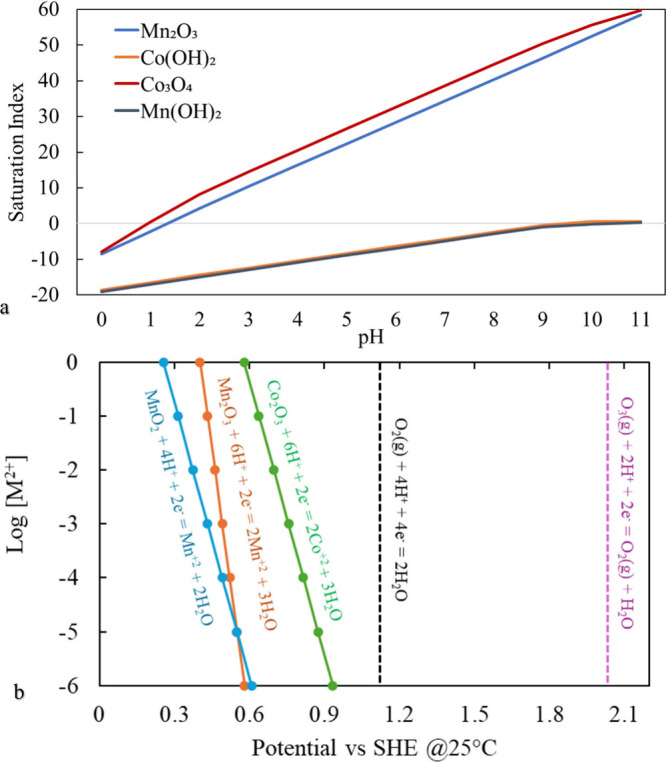
Saturation indexes of Co and Mn as a function of solution pH (a), and Eh–Log­[M^2+^] diagram for Co–Mn precipitation at pH 7 and 25 °C (b).

### 3.2 Parametric Study

To maximize the recovery of Co–Mn from aqueous solutions, the effect of key process variables, namely, stirring rate, flow rate, ion concentration, and temperature, on the ozone oxidative precipitation of these elements was systematically investigated. A combination of individual experiments and a statistically designed Box-Behnken study was employed to evaluate the effects and interactions of these parameters. The Box-Behnken design focused on the gas flow rate, temperature, and stirring speed to identify optimal operating conditions for oxidative precipitation using ozone.

#### 3.2.1 Effect of Gas Flow Rate

The gas flow rate, corresponding to the oxygen feed rate to the ozone generator, plays a critical role in the ozone oxidative precipitation of Co and Mn.^27^ It directly influences the amount of ozone available for redox reactions and the efficiency of gas–liquid mass transfer. To evaluate its effect, experiments were performed at room temperature (25 °C), neutral pH (7), and a fixed stirring speed of 400 rpm. Figure 4 presents the recovery trends of Co and Mn as a function of reaction time at different gas flow rates ranging from 200 to 2000 cc/min.

**Figure 4:**
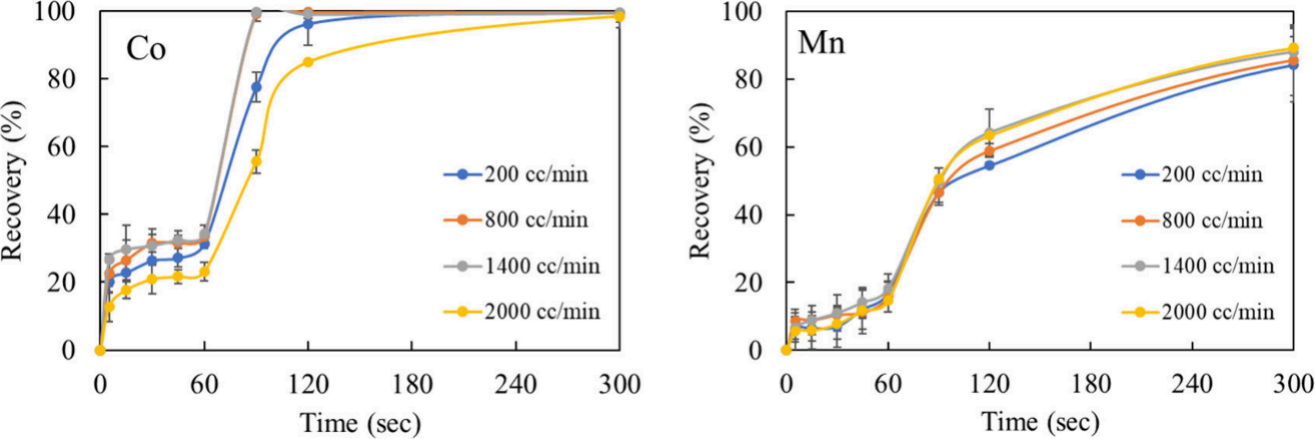
Co–Mn recovery values as a function of time at different gas flow rates.

The results show that Co was rapidly and nearly completely precipitated within 90 s, particularly at intermediate flow rates (800–1400 cc/min), where ozone dissolution and mass transfer were most effective. Interestingly, the Co recovery slightly decreased at the highest tested flow rate (2000 cc/min), which is likely due to a combination of incomplete ozone formation, accelerated ozone decomposition, and gas–liquid mass-transfer constraints at excessive gas flow rates. This behavior is supported by the reactions in the Supporting Information (Section S1.1.2), which explain that high gas flow rates lead to increased ozone decomposition in the ozone generator, where turbulence and gas–liquid interaction reduce ozone availability for the metal oxidation reaction (see reactions RS7-RS8 in Supporting Information).^42^ In addition, excessive gas flow through the liquid phase can decrease ozone residence time in the water and promote bubble collision and coalescence, producing larger bubbles with lower specific interfacial area. Consequently, a greater fraction of ozone may escape as off-gas before dissolving or reacting with dissolved Co. Together, these effects can reduce effective ozone utilization and explain the slight decrease in Co recovery observed at 2000 cc/min.

In contrast, Mn precipitation followed a slower kinetic profile, requiring more than 300 s to reach comparable recovery levels. The effect of flow rate on Mn recovery was less pronounced. This suggests that the ORP of the system remained within the range, facilitating rapid Mn oxidation (∼700 mV). Although Mn has a lower redox potential and is thermodynamically more susceptible to oxidation than Co (Figure 3b), the experimental results show that Co precipitates more rapidly during ozone treatment. While Mn^2+^ is oxidized more readily, the resulting Mn^3+^ species form less supersaturated phases compared to their Co counterparts. As shown in the saturation index diagram (Figure 3a), Co_3_O_4_ reaches significantly higher SI values than Mn_2_O_3_ at equivalent pH conditions, indicating a greater thermodynamic driving force for Co precipitation. This high supersaturation likely accelerates the nucleation and growth of Co oxide solids, leading to faster removal from the solution. Therefore, this behavior reflects stronger precipitation thermodynamics for Co, which outweigh the oxidation kinetics under the tested conditions.

#### 3.2.2 Effect of Stirring Speed

The stirring rate affects the mass transfer of ozone through mixing of the reaction system, thereby impacting the ozone oxidative precipitation process.^27^ Therefore, the effect of stirring rate on precipitation of Co and Mn using ozone was studied at room temperature (25 °C), neutral solution pH, and gas flow rate of 250 cc/min (Figure 5). Increasing stirring speed from 0 to 1200 rpm significantly improved Co–Mn precipitation in the first 90 s. This enhancement is attributed to improved dispersion of ozone in the solution, leading to increased contact between ozone and metal ions, and therefore accelerating their oxidation and subsequent precipitation. However, when the stirring rate was increased from 1200 rpm to 1500 rpm, a slight decrease in Co recovery was observed. This suggests that high agitation may promote local supersaturation and secondary nucleation, limiting crystal growth through reduced residence time and localized depletion of ozone or Co^2+^ near nucleation sites.^23^ Mn precipitation, being slower, less prone to overnucleation phenomena, and more diffusion-controlled, is less affected by these local fluctuations, resulting in more stable recovery across stirring speeds.

**Figure 5:**
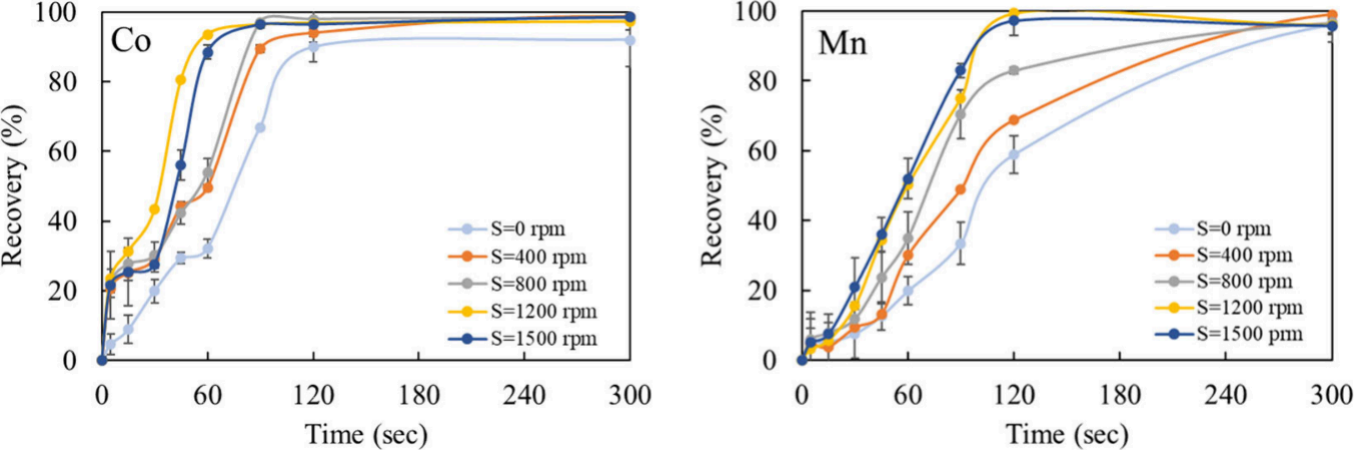
Co–Mn recovery values as a function of time at different stirring rates.

#### 3.2.3 Effect of Initial Co–Mn Concentration

The effect of initial ion concentration on the oxidative precipitation of Co and Mn was investigated. The experimental conditions were room temperature of 25 °C, pH 7, stirring speed of 1500 rpm, and gas flow rate of 1400 cc/min. As shown in Figure 6, increasing the initial concentration from 5 to 5000 ppm significantly affects the precipitation rate for both Co and Mn. At low concentration (5 ppm), both metals were rapidly and completely precipitated within about 1 min. Interestingly, Mn showed slightly faster precipitation kinetics than Co in this dilute case. This observation may be attributed to Mn^2+^ possessing a lower standard reduction potential (∼700 mV for Mn^3+^/Mn^2+^ vs 900 mV for Co^3+^/Co^2+^), making Mn more thermodynamically favorable to oxidize under dilute conditions. Additionally, at such low concentrations, mass transfer limitations are minimal, and ozone is present in stoichiometric excess, favoring rapid reaction kinetics for both metals. However, as the initial concentration increased, distinct differences in precipitation behavior emerged. At 50 ppm and above, Co consistently precipitated more rapidly than Mn. For example, at 5000 ppm, complete Co recovery was achieved within 2 h, while Mn required over 4 h to reach comparable recovery. As indicated by SI analysis (Figure 3a), Co oxides (Co_3_O_4_) exhibit higher supersaturation levels than Mn oxides (Mn_2_O_3_) under the same pH and ozone conditions, providing a greater thermodynamic driving force for Co precipitation. Higher supersaturation can lead to more rapid nucleation and crystal growth, particularly when supported by favorable ozone diffusion and sufficient residence time. Furthermore, Co oxides may form denser, more crystalline particles, thereby having lower surface energy, facilitating sustained precipitation, whereas Mn oxides tend to be poorly crystalline.^43,44^ Despite the variation in kinetics, the final recovery for both Co and Mn approached 100% across all concentrations (Figure 6), indicating that concentration-dependent effects primarily govern the kinetics rather than being limited to reaction completion. These findings are consistent with prior studies,^27,28^ which demonstrated that, although both metals can be efficiently recovered using ozone, their precipitation pathways differ in kinetics and supersaturation behavior.

**Figure 6:**
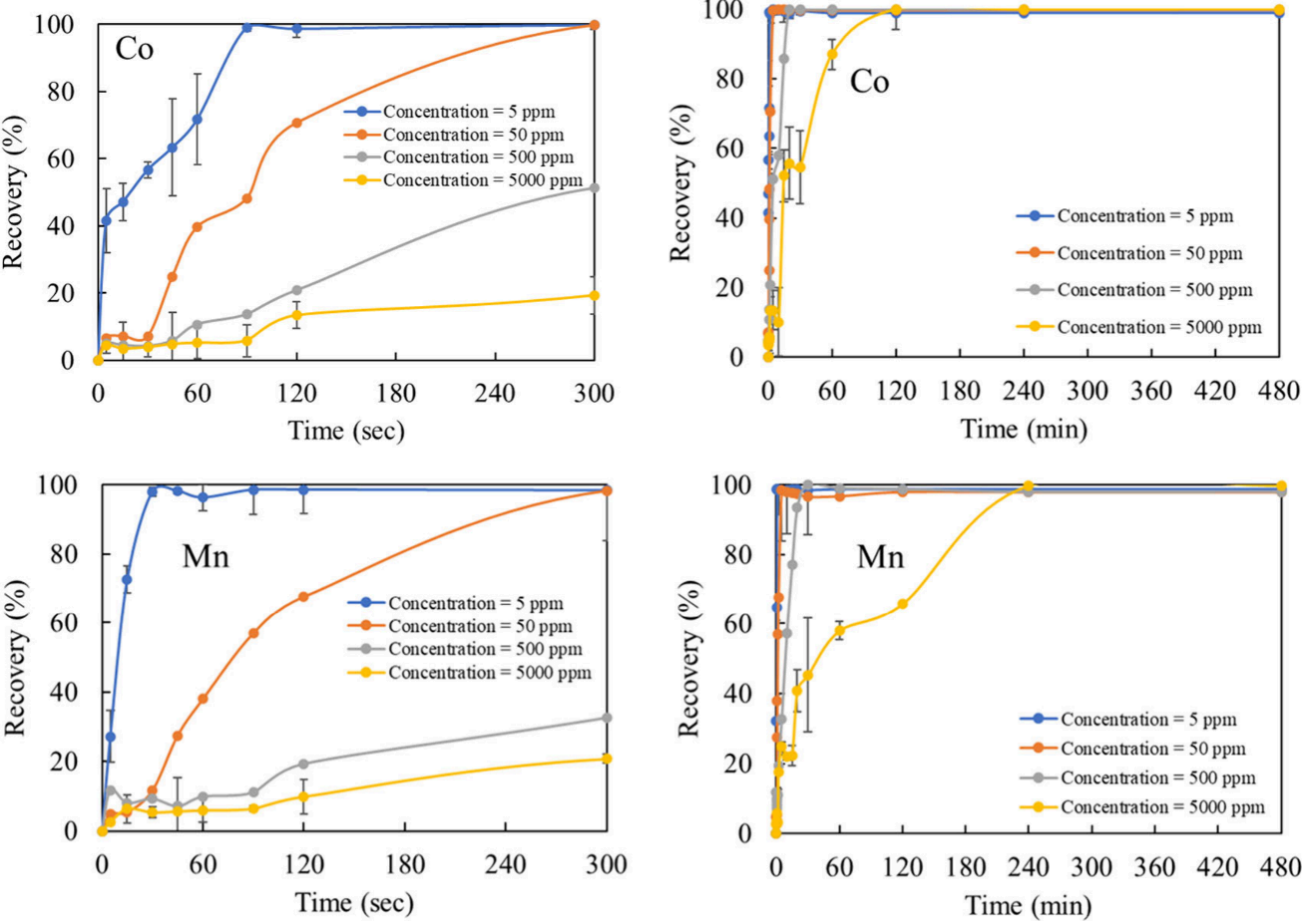
Co–Mn recovery values as a function of time at different initial ion concentrations.

#### 3.2.4 Effect of Temperature

Temperature plays a critical role in ozone oxidative precipitation processes, influencing both reaction kinetics and gas–liquid mass transfer characteristics.^21,22,27^ The effect of temperature on the oxidation of Co and Mn precipitation was evaluated using the prepared stock solutions at neutral pH, a stirring rate of 400 rpm, and a gas flow rate of 1100 cc/min (Figure 7). Kinetic data were analyzed using three different models, with the pseudohomogeneous model providing the best fit, accounting for both homogeneous and mass-transfer limitations. The activation energy was measured using eq (S7) in Supporting Information to understand the mechanism governing the ozone oxidative precipitation of Co and Mn. Figure 7 shows that oxidative precipitation proceeds rapidly, with more than 50% of Co and Mn removed from solution within the first 90 s across all temperatures. Co recovery initially increased from 20 °C to 40 °C, suggesting enhanced ozone mass transfer and reactivity, but declined at 80 °C, likely due to increased ozone decomposition and reduced solubility at elevated temperatures. In contrast, Mn recovery increased significantly within the first minute, after which it became less sensitive to temperature. This behavior suggests that the ORP of the system, which exceeded ∼ 700 mV, remained sufficient for Mn^2+^ oxidation despite diminished ozone availability.

**Figure 7:**
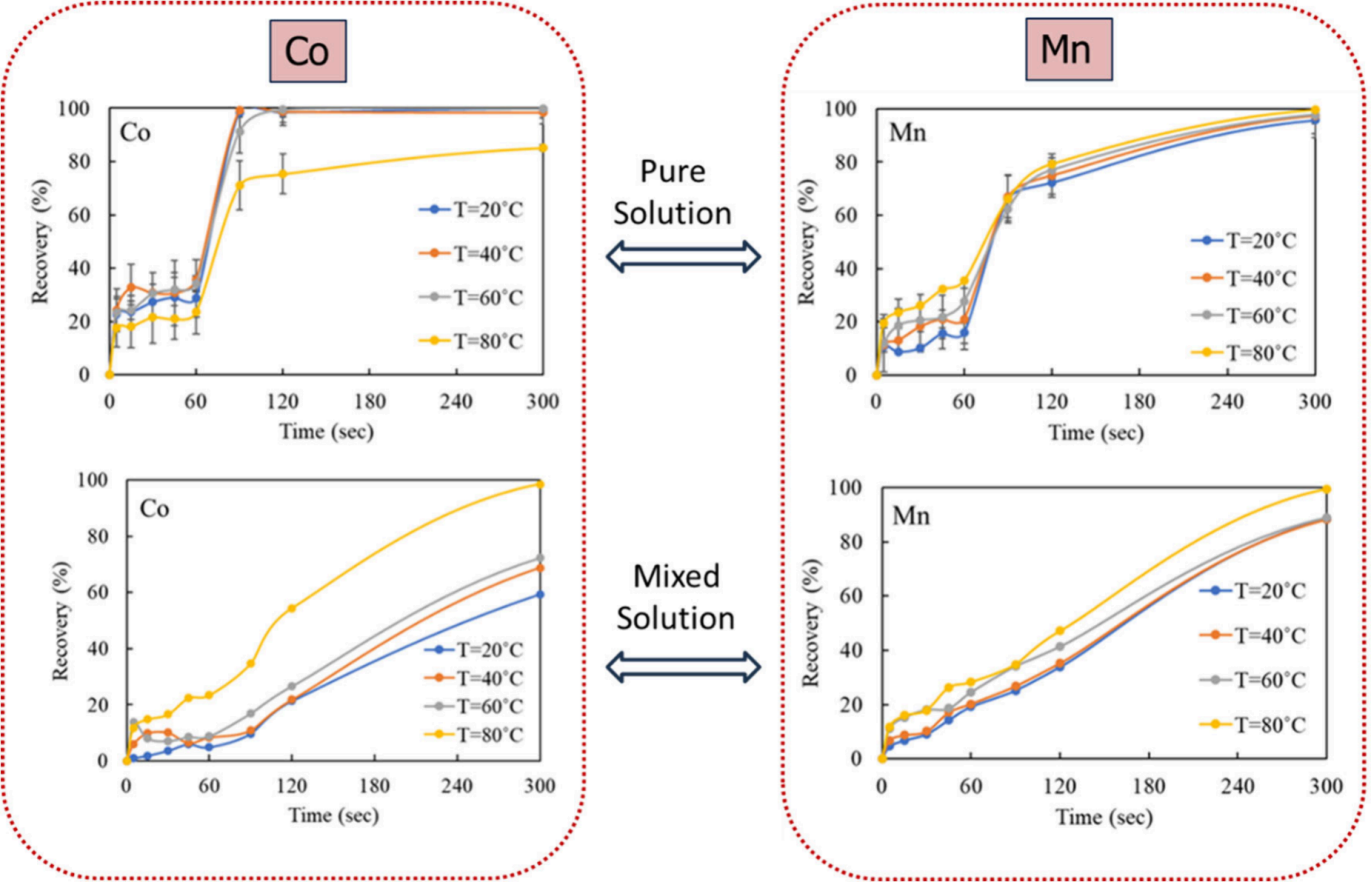
Effect of temperature (20, 40, 60, and 80 °C) on Co–Mn precipitation in pure and mixed Co–Mn solutions.

In a separate set of experiments with a mixed Co–Mn solution, containing the same concentrations that were used in the pure Co and Mn solutions and conducted under the same conditions, both Co and Mn recovery improved steadily with increasing temperature (Figure 7), opposite to the trend observed in the pure Co system. This divergence suggests a synergistic effect, where Mn oxides such as MnO_2_ or MnOOH serve as reactive surfaces for Co^2+^ adsorption or facilitate its oxidative coprecipitation. These results support the hypothesis that Mn oxides, once formed, enhance Co removal via surface complexation and redox interactions even under thermally limited ozone conditions.^45^

Coprecipitation refers to the simultaneous precipitation of multiple species in a solution. This phenomenon often involves the incorporation of foreign ions into the crystal lattice of a growing phase via chemisorption or substitution mechanisms, especially when these ions share similar size and charge characteristics^46^ (Figure 8). In this study, fine Co nuclei, formed in earlier stages, appeared to not aggregate to form large, sedimentable particles. Instead, Co precipitates, ranging from submicrometer to micrometer in size, likely adsorbed onto the surfaces of larger, rapidly forming Mn precipitates and subsequently co-settled with them.^47–49^ The interaction was corroborated by precipitate characterization.

**Figure 8:**
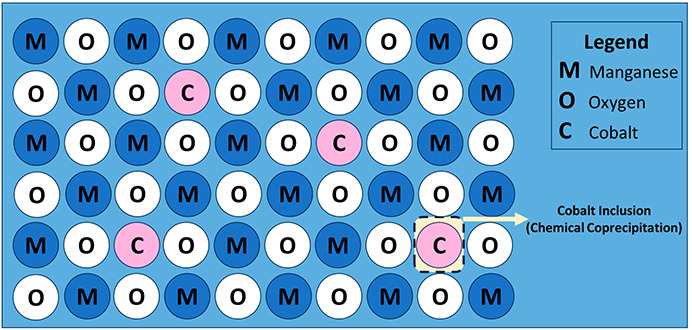
Schematic of coprecipitation for a chemically adsorbed inclusion within a crystal lattice, where M (Manganese) and O (Oxygen) represent the cation–anion pair comprising the analyte and the precipitant, and C denotes a secondary cation (i.e., Co) that becomes incorporated into or substituted within the growing Mn oxide phase.

Precipitates were analyzed using SEM-EDS, XRD, XPS, Zetasizer, and FTIR to investigate their morphology, formation, surface chemistry, and structure, including those from pure Co and Mn and mixed Co–Mn solutions (Figures 9–12). Zeta potential analysis provided compelling evidence for Co adsorption onto Mn oxides. Precipitates of pure Mn and Co solutions exhibited distinct surface charges (−36.8 mV and – 4.5 mV, respectively), whereas the precipitate of a mixed solution displayed an even more negative potential (−40.6 mV), indicating enhanced colloidal stability and increased surface reactivity (Figure 9). The negative charge on freshly precipitated MnO_2_ particles promotes electrostatic attraction of Co^2+^ ions, facilitating their immobilization.

**Figure 9:**
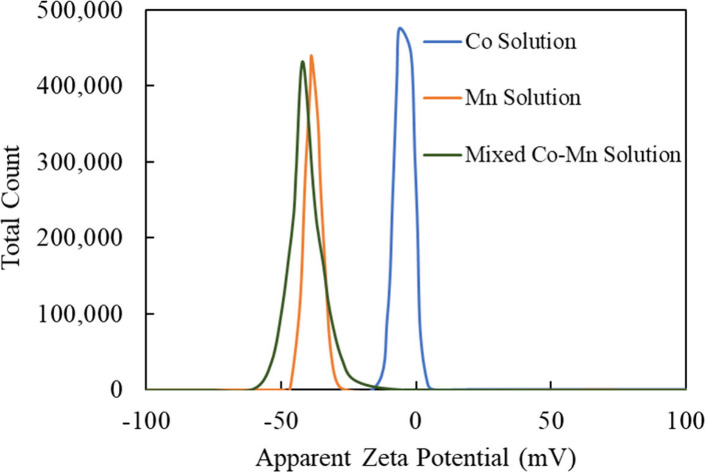
Surface charge (zeta potential) (mV) of precipitates obtained from ozone oxidative precipitation of pure Co, pure Mn, and mixed Co–Mn solutions at pH 7.

**Figure 10:**
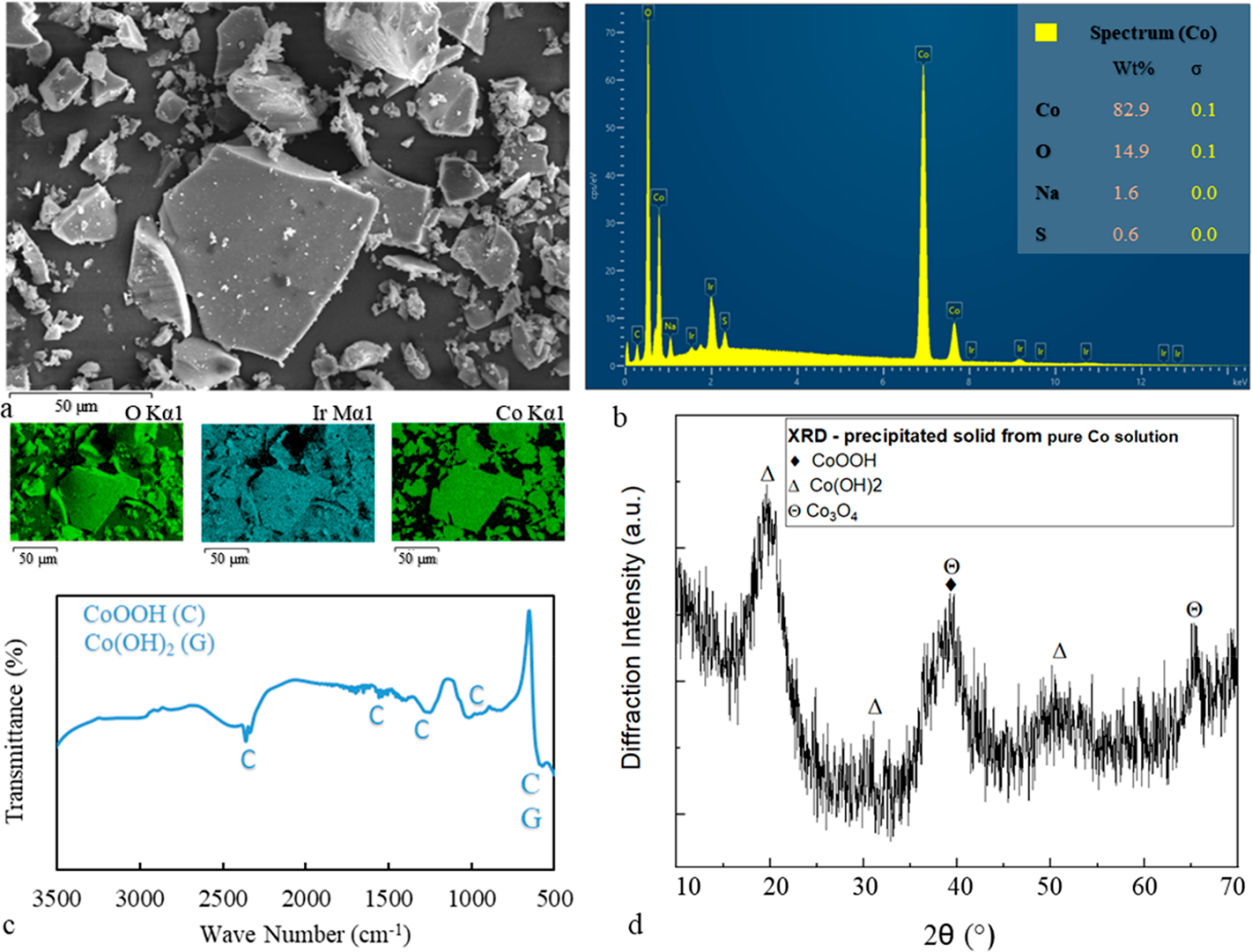
SEM micrographs (a), EDS spectrum (b), FT-IR spectrum (c), and XRD pattern (d) of precipitated solids from oxidative ozone precipitation for pure Co solution.

**11:**
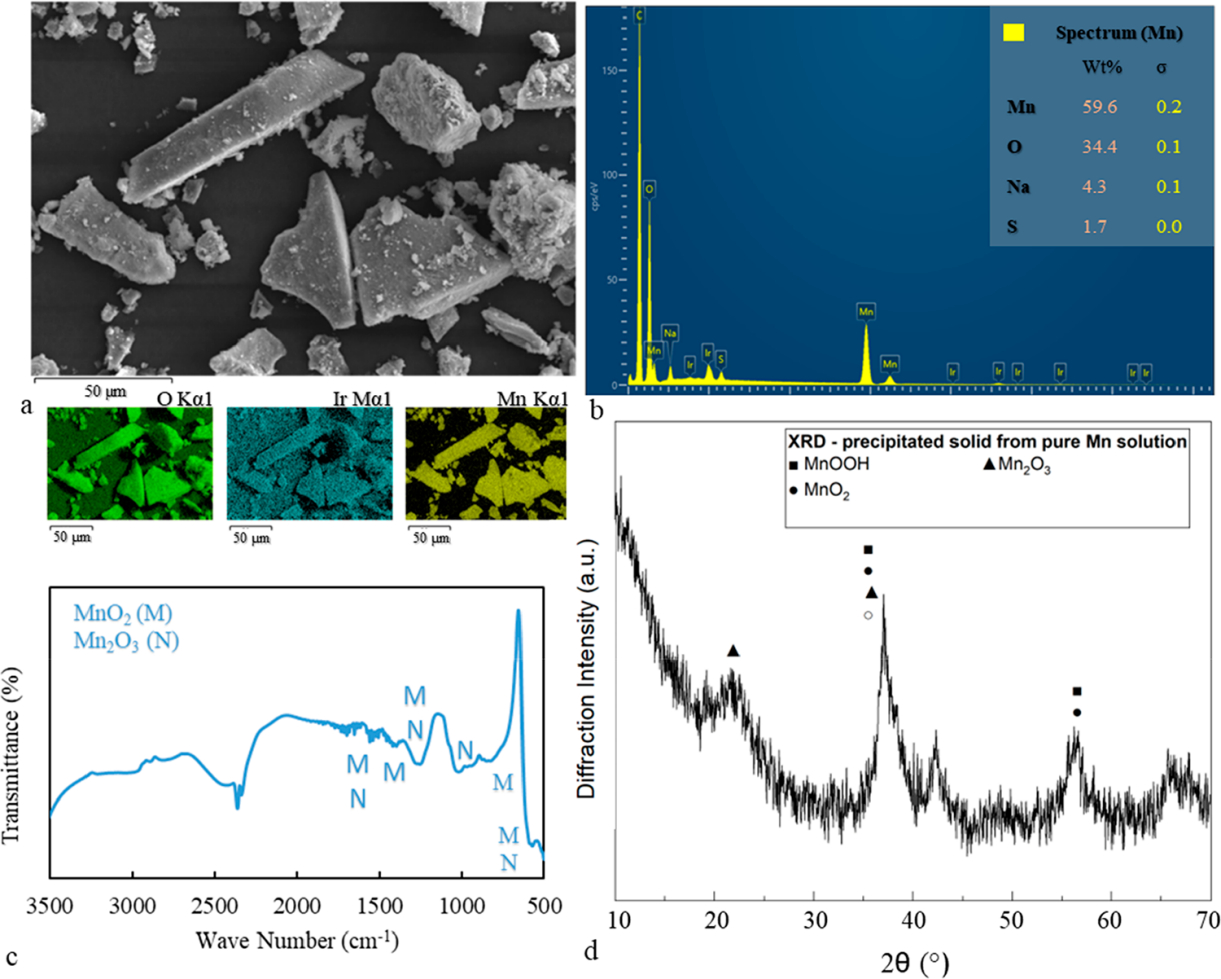
SEM micrographs (a), EDS spectrum (b), FT-IR spectrum (c), and XRD pattern (d) of precipitated solids from oxidative ozone precipitation for pure Mn solution.

**Figure 12:**
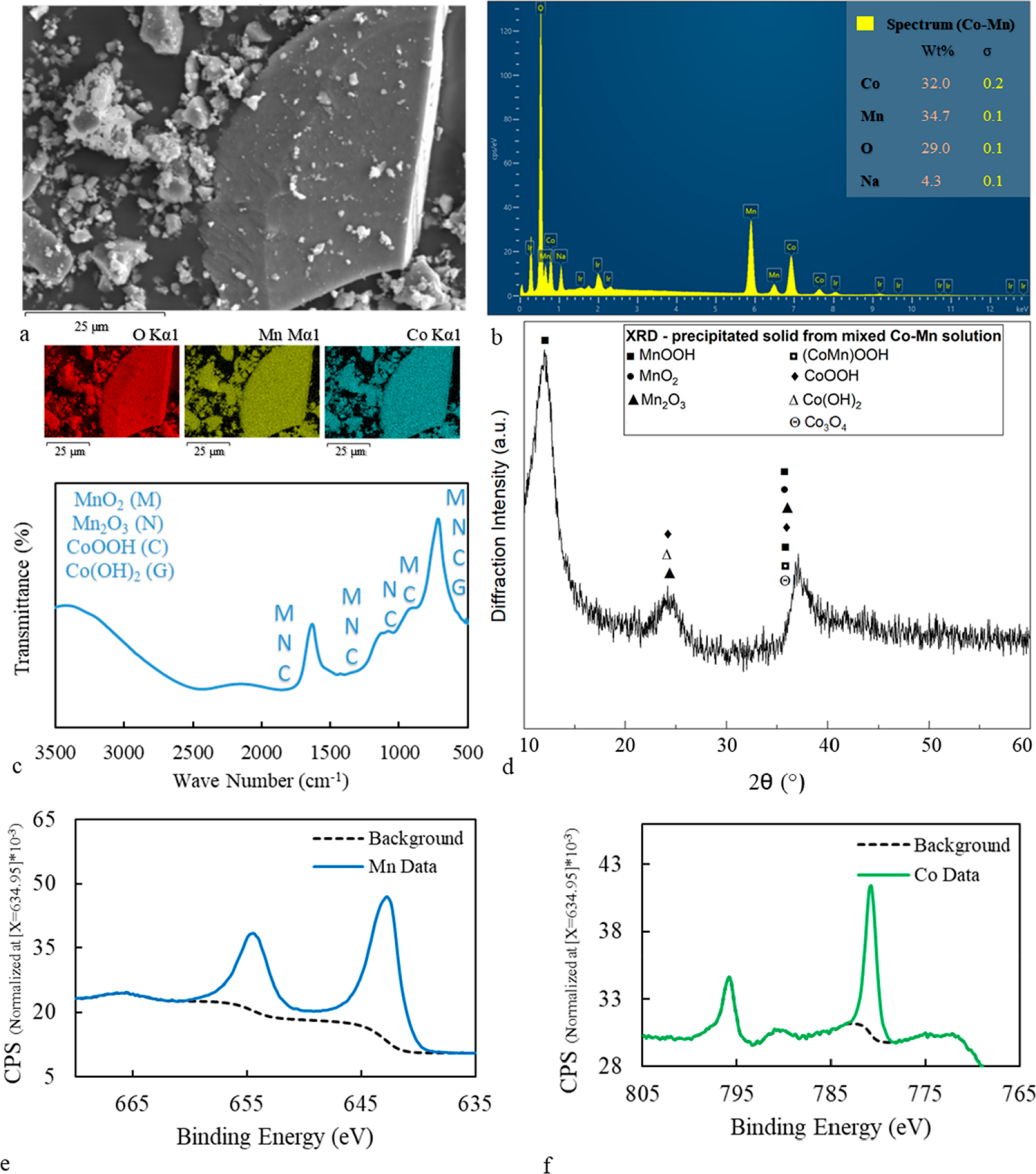
SEM micrographs (a), EDS spectrum (b), FT-IR spectrum (c), XRD pattern (d), XPS spectra for Mn (e) and Co (f) of precipitated solids from oxidative ozone precipitation for pure mixed Co–Mn solution.

The enhanced Co recovery observed at elevated temperatures in the mixed solution, contrasting with its decline in the pure Co system, therefore results from increased adsorption and subsequent oxidative coprecipitation of Co on MnO_2_. These findings align with the mechanism proposed by Hem et al.,^45^ in which Co^2+^ is oxidized at the surface of Mn oxide particles to form CoOOH, particularly when Mn^3+^/Mn^4+^ oxides act as electron mediators. In systems with continuous oxidant input, such as ozone purging, MnO_2_ functions as both a product of Mn oxidation and a reactive surface that adsorbs and oxidizes Co^2+^. This process may be further intensified by localized supersaturation and elevated ligand concentration near MnO_2_ surfaces, creating microenvironments that enhance Co incorporation via coprecipitation or surface complexation mechanisms. The more negative zeta potential observed in the precipitates of the mixed Co–Mn solution reinforces the presence of stable, highly reactive Mn oxide particles with high affinity for Co^2+^.

The SEM-EDS images revealed that most of the Co–Mn precipitated particles were in the submicron to supramicron size range, exhibiting irregular morphologies with rough surfaces. XRD analysis of precipitates formed from pure Co, pure Mn, and mixed Co–Mn solutions revealed that Mn and Co precipitates were in the forms of manganese oxide (MnO_2_), manganese oxyhydroxide (MnOOH), dimanganese trioxide (Mn_2_O_3_), cobalt oxyhydroxide (CoOOH), cobalt hydroxide (Co­(OH)_2_), cobalt­(II,III) oxide (Co_3_O_4_), and cobalt–manganese oxyhydroxide ((Co–Mn)­OOH)). However, some reflections were broad and partially overlapped, indicating limited crystallinity and the possible presence of amorphous components. Such features are typical for solids obtained from ozone oxidative precipitation, which often exhibit short-range structural order rather than fully developed crystallinity.^21,33^ Therefore, complementary characterization techniques such as FT-IR and XPS were used to verify the chemical composition and oxidation states of the Co–Mn solids. These mineralogical phases were further confirmed by the FT-IR analysis (Figures 10–12). The FT-IR spectra showed a strong absorption band at around 528, 1049, and 1283 cm^–1^, corresponding to vibrations of (Co,Mn)-O and (Co,Mn)–OH bond in the MnO_2_, Mn_3_O_4_, and CoOOH structures.

XPS measurements were performed on precipitates obtained from the mixed Co–Mn solution to determine the surface oxidation states of manganese and cobalt (Figure 12). The Mn 2p spectrum displayed two distinct peaks at binding energies of approximately 641.9 eV (Mn 2p_3/2_) and 653.5 eV (Mn 2p_1/2_), consistent with the presence of Mn^4+^, typically associated with MnO_2_. The absence of significant peak shifting or satellite features further confirms that Mn is predominantly present in the +4 oxidation state, indicative of strong oxidative conditions and effective Mn^2+^ conversion to MnO_2_. The Co 2p spectrum showed two main peaks at approximately 780.3 eV (Co 2p_3/2_) and 796.0 eV (Co 2p_1/2_), characteristic of Co^2+^, most likely in the form of Co­(OH)_2_. The spectrum also showed minor satellite structures, which are typical of high-spin Co^2+^ environments in hydroxide phases. Notably, the absence of a distinct shoulder near ∼ 779 eV suggests that Co^3+^ species such as CoOOH may be present only in small quantities or incorporated into mixed-phase structures, potentially obscured by dominant Co^2+^ signals.

To further differentiate weak surface adsorption from stronger association with the Mn oxide precipitates, selective leaching experiments were conducted by using 0.1 M HCl with sonication. Under these mild conditions, negligible Co release (<0.01%) was detected, whereas complete acid digestion quantitatively dissolved Co. These findings indicate that Co was not weakly surface-adsorbed but instead was strongly associated with the Mn oxide precipitates, consistent with coprecipitation and/or strong interfacial binding. XPS analysis showed Mn predominantly in the Mn­(IV) oxidation state and Co predominantly as Co­(II), supporting a surface-mediated coprecipitation mechanism and a strong association of Co with the Mn oxide phase.

Together, these results confirm that under ozone-driven oxidative conditions at neutral pH, Mn is rapidly and fully oxidized to MnO_2_, forming particles with rough, irregular morphologies and high surface energy. Such defect-rich Mn oxide surfaces provide abundant active sites for both adsorption and heterogeneous nucleation, locally concentrating Co^2+^ and facilitating its subsequent surface-mediated precipitation as Co­(OH)_2_ or CoOOH. The detection of mixed-phase cobalt–manganese oxyhydroxides ((CoMn)­OOH) in the XRD patterns indicates partial structural incorporation of Co into Mn oxide matrices. The absence of well-faceted cobalt-rich crystals further supports that Co precipitation in mixed systems is driven primarily by adsorption and coprecipitation on Mn oxide surfaces, where high surface energy accelerates nucleation but may limit subsequent crystal growth. In this way, Mn acts not only as a primary precipitate but also as a redox-active substrate promoting Co immobilization through both physical and chemical interactions.

These temperature-dependent precipitation results highlight the critical role of ozone availability in the oxidative removal of Co and Mn. While elevated temperatures enhance molecular activity and reaction kinetics, they simultaneously reduce ozone solubility and promote its decomposition, therefore limiting the effective oxidant concentration in solution and its application for high-temperature solutions.^50,51^ This dual influence necessitates careful optimization of process parameters to balance physical mass transfer enhancements with chemical oxidant stability. This is especially critical when considering this process for medium-temperature applications. The rapid precipitation observed within the first 120 s of ozone addition at neutral pH (Figure 7) suggests that the reaction is kinetically favorable, driven by ozone’s high oxidative potential. According to the Arrhenius relationship, increasing temperature boosts molecular kinetic energy and reaction rate. Additionally, elevated levels of oxidants in a supersaturated solution accelerate nucleation by altering the equilibrium state, corroborating previous findings that high supersaturation promotes rapid precipitation.^48,52^ However, further increases in temperature reduce ozone solubility (Figure S4 in Supporting Information) and accelerate its decomposition, limiting the oxidant available for Co precipitation. Ozone decomposition occurs through the following reactions ([Disp-formula eqR1]-[Disp-formula eqR6]):

R1 $$O_{3}\to O_{2}+O$$

In the direct decomposition of ozone (R1), monatomic oxygen (O), which is highly reactive, can be formed as an intermediate in the decomposition of ozone. However, due to its high reactivity, it frequently engages in subsequent reactions:

R2 $$O+O_{3}\to 2O$$

R3 $$O+H_{2}O\to 2{\mathrm{OH}}^{•}$$

At near-neutral to basic pH, ozone can also decompose through pathways involving hydroxyl radical formation:

R4 $$O_{3}+H_{2}O\to 2{\mathrm{OH}}^{•}+O_{2}$$

R5 $${\mathrm{OH}}^{•}+O_{3}\to {{\mathrm{HO}}_{2}}^{•}+O_{2}$$

R6 $$H{O_{2}}^{•}+O_{3}\to {\mathrm{OH}}^{•}+2O_{2}$$

At neutral pH, hydroxyl radical-mediated chain reactions accelerate ozone decomposition, reducing effective oxidant availability and limiting Co precipitation at elevated temperatures.^48,50,51,53^

To investigate these effects quantitatively, the volumetric mass transfer coefficient, K_L_a, was determined experimentally under varying temperatures and gas flow rates and compared with theoretical predictions. K_L_a is governed by both gas–liquid interfacial dynamics and physicochemical parameters such as temperature, gas holdup, and superficial gas velocity.

As shown in Figure 13a, theoretical K_L_a values increased consistently with temperature due to enhanced ozone diffusivity and lower kinematic viscosity of the liquid, both of which improve mass transfer rates. In contrast, experimental K_L_a initially increased from 20 °C (0.0032 s^–1^) to 40 °C (0.0057 s^–1^) but decreased sharply to 0.0022 s^–1^ at 60 °C and 0.0003 s^–1^ at 80 °C. This discrepancy arises from reduced ozone solubility and accelerated decomposition above 40 °C, which offset gains from enhanced mass transfer^47^ (Figure 13a; Figure S4 in Supporting Information).

**Figure 13:**
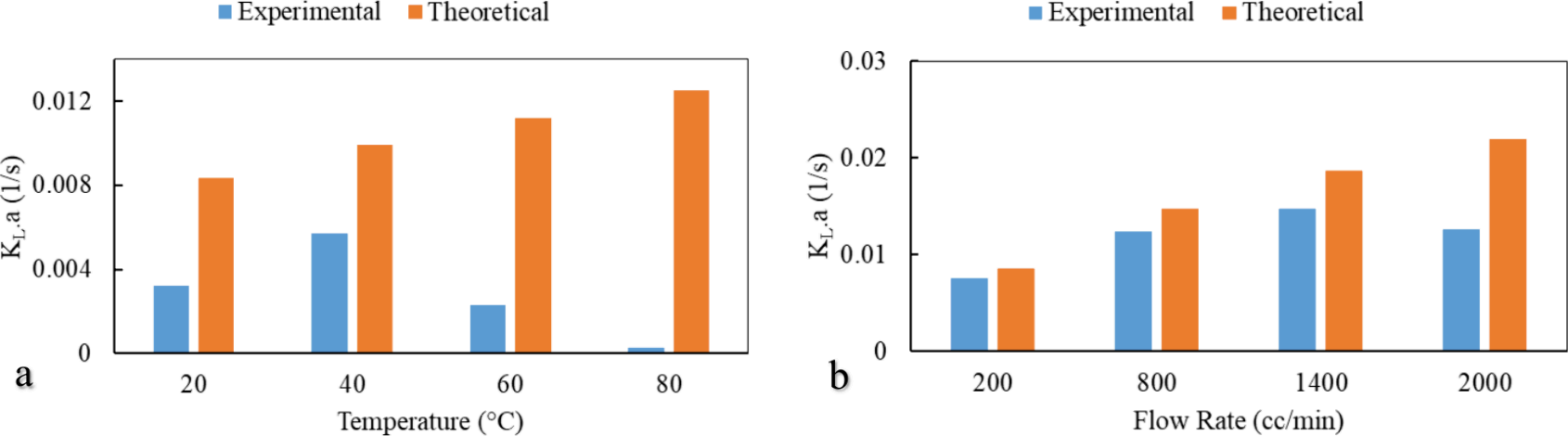
Experimental vs theoretical volumetric mass transfer coefficients (K_L_a) at different (a) temperatures and (b) flow rates.

In addition to temperature, the influence of gas flow rate was also investigated. As shown in Figure 13b, increasing the ozone–oxygen gas flow rate from 200 to 1400 cc/min resulted in a significant increase in experimental K_L_a, from 0.0075 s^–1^ to 0.0147 s^–1^, attributed to enhanced ozone availability and improved bubble dispersion (eq (S1−S3) in Supporting Information). This trend is consistent with studies published by Bouaifi et al.,^54^ who demonstrated that K_L_a increases with gas flow due to increased superficial velocity (U_G_) and interfacial area (a). However, at 2000 cc/min, a slight decline in K_L_a to 0.01265 s^–1^ was observed, attributed to the reduced residence time of oxygen inside the ozone generator, which results in incomplete ozone formation. Similar findings from Ratnawati et al.^40^ noted that excessive gas flow can dilute or thermally decompose ozone before reaching the reactor, therefore decreasing effective ozone concentration and K_L_a.^40^

At moderate conditions (Temperature: 40 °C and flow rate: 800 cc/min), the experimentally determined volumetric mass-transfer coefficient from the two-film model (K_L_a ≈ 0.0057 s^–1^) corresponds to a characteristic ozone diffusion time of t ≈ 175 s (1/K_L_a). This value is in close agreement with the kinetic time scale derived from the pseudohomogeneous model (t ≈ 135 s (1/k″)). The comparable magnitude of these characteristic times indicates that ozone mass transfer and oxidation–precipitation reactions occur on similar time scales, demonstrating that oxidant transport directly constrains the observed precipitation rate. Therefore, variations in K_L_a under different operating conditions are expected to translate into proportional changes in effective oxidant availability and overall precipitation kinetics. This similarity indicates that neither mass transfer nor reaction kinetics exclusively dominate the process. Instead, the overall precipitation rate is governed by the coupling of ozone transfer across the gas–liquid interface and the subsequent oxidation–precipitation reactions occurring at the solid–liquid interface. Together, these findings highlight the importance of carefully balancing temperature and gas flow to ensure sufficient ozone generation, dissolution, and chemical reactivity. In systems like ozone-driven oxidative precipitation, where rapid kinetics and supersaturation are key, both physical mass transfer and oxidant stability must be simultaneously optimized to achieve adequate precipitation of target metals.

The differences in precipitation behavior of Mn and Co with respect to temperature may stem from a combination of factors, including differences in solubility, redox chemistry, ion concentrations, and solution composition. Manganese oxides generally exhibit lower solubility at higher temperatures, which favors solid-phase formation. As temperature rises, the solubility product of Mn oxides such as MnO_2_ and MnOOH decreases, shifting the equilibrium toward precipitation and reducing the concentration of dissolved Mn ions in solution.^55^ Moreover, although ozone solubility and stability decrease at elevated temperatures, Mn oxidation was not significantly hindered. This suggests that the ORP of the system (∼700 mV) remained within a favorable range for Mn^2+^ to MnO_2_ conversion, even under reduced ozone availability.^33^ Furthermore, Mn oxidation may proceed more efficiently due to favorable redox kinetics and a potentially lower activation energy, allowing the reaction to proceed effectively despite diminished oxidant concentration.

Further observations revealed that after the initial minutes of reaction, the precipitation of Co and Mn initially slightly decreased and then gradually increased over the duration of the experiments. This behavior is consistent with Ostwald ripening, in which smaller, less stable particles dissolve and redeposit onto larger ones.^48,52^ It is important to highlight that while the precipitation was visibly significant for up to 5 min, it continued until the experiment concluded in 30 min.

The distinctions in Co and Mn precipitation from the prepared mixed solutions were further evaluated under optimal operating conditions achieved from the effect of individual parameters (80 °C, 1400 cc/min flow rate, and 1500 rpm stirring rate). As shown in Figure S5 in Supporting Information, both metals reached a near-complete recovery within the first 2 min, confirming the fast kinetics of the oxidative precipitation process.

In this study, kinetic rates and apparent activation energies were evaluated during the initial 30 s using Linear, Higbie, and Pseudohomogeneous models. The Pseudohomogeneous model provided the best fit (Figures S6–S7 in Supporting Information), suggesting that the oxidative precipitation process is influenced by both homogeneous reaction kinetics and mass transfer limitations. The rate of Mn precipitation was faster than that of Co in both pure (Mn: 0.0107 s^–1^; Co: 0.0088 s^–1^) and mixed (Mn: 0.0062 s^–1^; Co: 0.0011 s^–1^) solutions. This suggests that Mn oxidizes and nucleates more readily than Co under identical conditions, likely due to its lower redox potential and more favorable precipitation kinetics. The calculated apparent activation energies further support this conclusion. For pure Co and Mn in pure solutions, the activation energies were determined as Ea_(Co)_ = 8.6 kJ/mol and Ea_(Mn)_ = −15.3 kJ/mol, while in mixed Co–Mn solution, values increased to Ea_(Co)_ = 11.4 kJ/mol, and Ea_(Mn)_ = −12.3 kJ/mol. The activation energies reported in this study (Figure 14) represent apparent activation energies derived from experimentally observed precipitation rates and therefore reflect the combined influence of ozone mass transfer, ozone solubility, decomposition, and intrinsic oxidation kinetics. Because ozone becomes less soluble and decomposes more rapidly at higher temperatures, its effective concentration in solution does not change monotonically with temperature. As a result, the low or negative apparent activation energies likely arise from constraints imposed by oxidant availability and mass-transfer effects, rather than from the intrinsic activation barrier of the metal oxidation reaction itself. Under the investigated conditions, the oxidative precipitation of Co and Mn is more appropriately described as being governed, or at least strongly influenced, by ozone mass transfer rather than solely by intrinsic reaction kinetics. These results further indicate that ozone transport and aqueous stability play a governing role in determining the overall precipitation kinetics. Moreover, the increased activation energy for cobalt in mixed systems reinforces the hypothesis that Mn oxides act as catalytic or reactive surfaces facilitating Co^2+^ removal via surface interactions, especially under conditions where ozone availability alone is insufficient.

**Figure 14:**
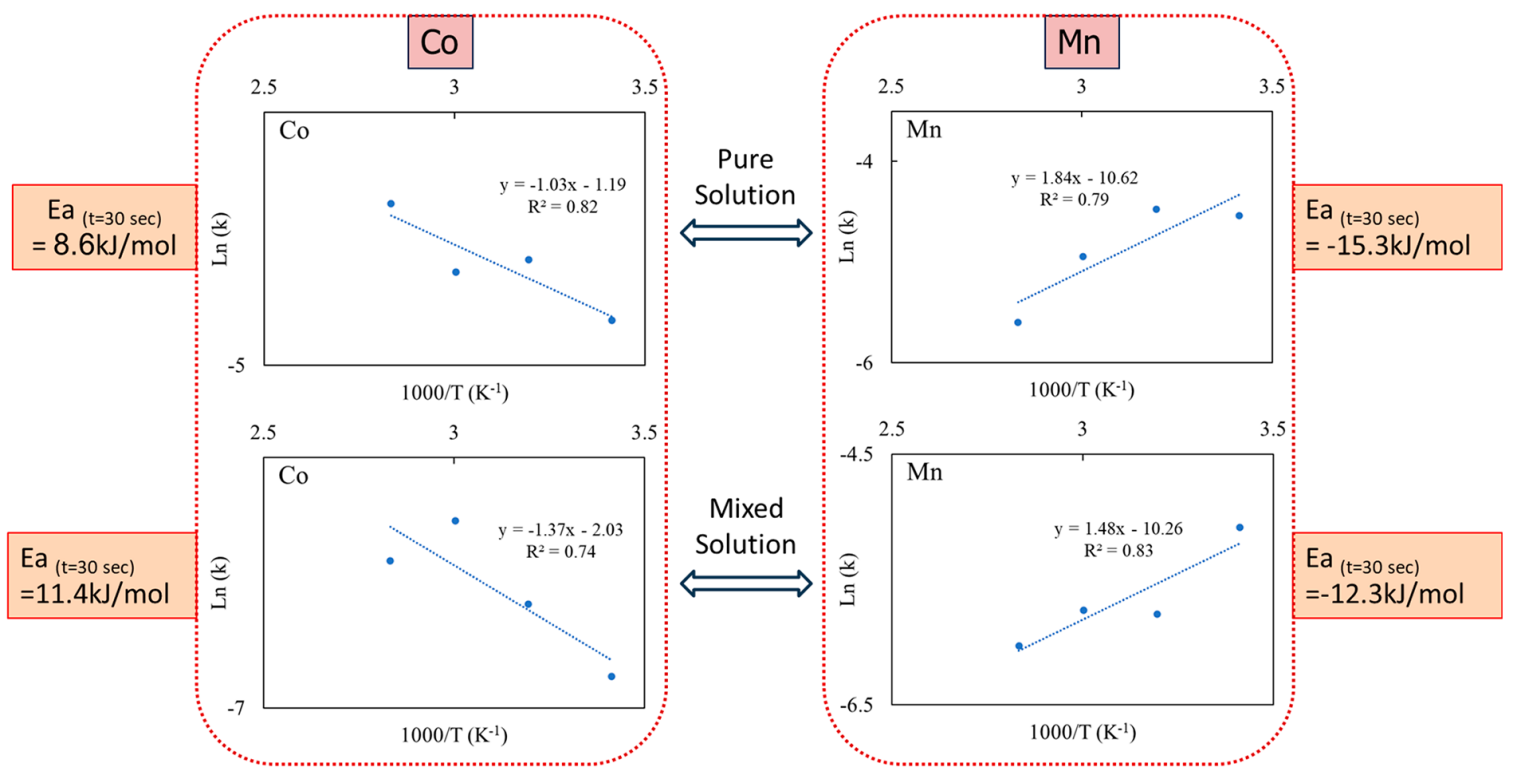
Activation energy for Co and Mn oxidative precipitation in pure and mixed systems.

To further evaluate the combined effects of the main operating parameters, a three-level Box–Behnken statistical design was applied to analyze the individual and interactive effects of temperature, gas flow rate, and stirring rate on Co–Mn recovery during the initial 30 s precipitation window used for kinetic modeling. The key ANOVA results and response-surface plots for Co and Mn recovery are presented in Figures 15a and 15b, with the detailed parametric evaluation and supporting discussion provided in the Supporting Information (Section S2.2). Co recovery was most sensitive to temperature and gas flow rate, showing an optimum at moderate conditions, whereas Mn recovery increased steadily with both temperature and gas flow rate (Figures 15a and 15b). These trends are consistent with the kinetic behavior discussed earlier, reflecting the temperature dependence of ozone availability and the distinct oxidation pathways of Co and Mn.

**Figure 15:**
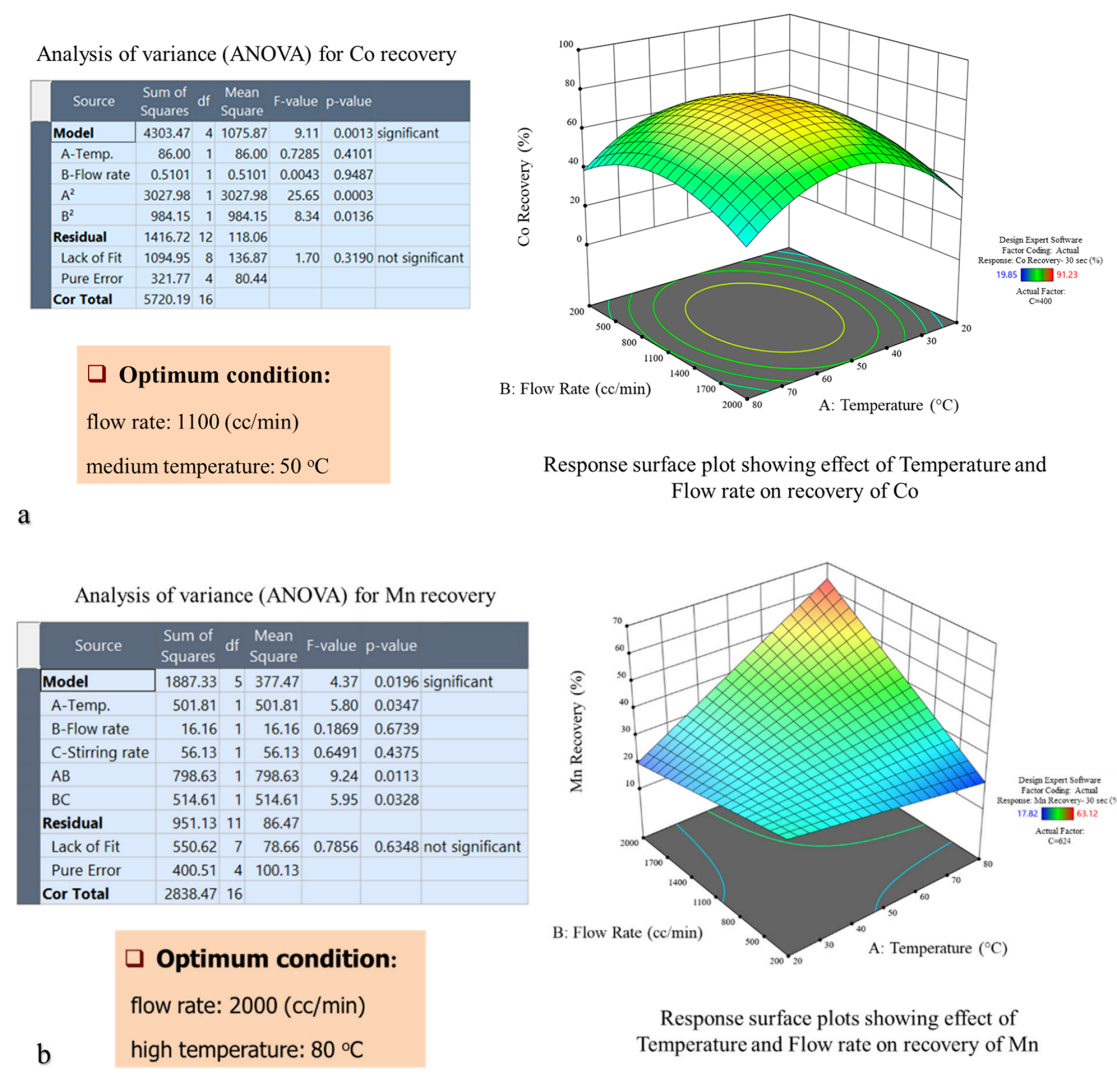
Statistical analysis and response surface plot for Co (a) and Mn (b) recovery as a function of significant process parameters.

Despite efforts to define a specific mechanistic pathway, the oxidative precipitation of Co and Mn via ozone is inherently complex and involves a series of concurrent phenomena. The process is initiated with the mass transfer of ozone from the gas to the liquid phase, governed by interfacial area, bubble dynamics, and liquid-phase diffusivity. Once dissolved, ozone must remain chemically stable for a sufficient period of time to react with metal ions. However, its stability is highly sensitive to temperature and pH and is susceptible to decomposition into molecular oxygen or forming hydroxyl radicals through radical chain pathways (see R1-R6). The competitive side reactions with water, other solution constituents, or organics, if present, may further scavenge ozone or hydroxyl radicals, reducing its effectiveness.

Following ozone dissolution, the system enters an early phase during which the physicochemical conditions necessary for nucleation and precipitation gradually develop. During this period, several processes may occur, including the oxidation of metal ions by ozone or its radical byproducts, local supersaturation around gas–liquid interfaces, and initiation of solid-phase formation. These steps are further complicated by the conversion of ozone to oxygen in side reactions, which do not directly contribute to redox precipitation. Moreover, the simultaneous presence of Co^2+^ and Mn^2+^ in solution introduces coprecipitation effects that alter surface chemistry and nucleation behavior. As Mn^2+^ rapidly oxidizes to MnO_2_ or MnOOH, it creates surfaces that can serve as adsorption or nucleation sites for cobalt. This results in surface-mediated reactions where Co^2+^ may be adsorbed, oxidized, or incorporated into mixed hydroxide phases.

In mixed-metal systems, heterogeneous nucleation dominates due to a lower Gibbs free energy barrier (ΔG_het_ < ΔG_hom_), facilitating faster and more efficient particle formation.^48,52^ Therefore, nucleation in these systems is more thermodynamically favored, facilitating faster and more efficient particle formation. Furthermore, once initial nuclei form, subsequent precipitation tends to occur through growth on existing surfaces rather than through formation of new nuclei, effectively suppressing secondary nucleation and promoting aggregation (Figure 16a). In contrast, homogeneous nucleation dominates in pure solutions, requiring higher supersaturation and leading to slower onset time and greater sensitivity to oxidant stability and diffusion (Figure 16b).

**Figure 16:**
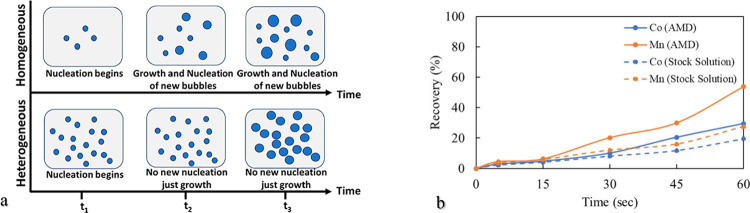
Comparison of nucleation behavior in homogeneous vs heterogeneous systems for Co and Mn precipitation in mixed-metal solutions (AMD) vs stock solutions (Figure 16a is conceptually adopted from Verdolotti et al.^56^).

Although Mn is thermodynamically easier to oxidize than Co, with a higher redox potential for Mn^2+^ → MnO_2_ compared to Co^2+^ → Co_3_O_4_, its precipitation is slower than Co. This is due to the higher nucleation barrier for Mn oxide formation, which requires a higher degree of supersaturation to form stable solid phases. In contrast, Co precipitates more readily due to a lower nucleation barrier, forming stable oxides at lower supersaturation levels. Therefore, while Mn oxidation is thermodynamically favorable, the precipitation of Mn oxides is slower, primarily due to the high nucleation energy required to form Mn oxide phases. At elevated temperatures, Mn oxides such as MnO_2_ and MnOOH act as nucleation sites for Co, accelerating its precipitation. This interaction further reconciles the apparent discrepancy between Mn’s easier oxidation and its slower precipitation kinetics.

Taken together, these results collectively demonstrate that Co–Mn oxidative precipitation is fundamentally diffusion-controlled, especially with respect to ozone mass transfer. In a real-world aqueous solution, the presence of pre-existing solids and catalytic impurities reduces the energy barrier for nucleation, stabilizes particle growth, and compensates for declining ozone availability at elevated temperatures. In contrast, synthetic or pure stock solutions are more sensitive to operational parameters such as temperature, gas flow, and mass transfer parameters due to the absence of these catalytic or impurities.

The experimentally observed K_L_a values (Figure 13) indicate that ozone transfer efficiency strongly influences oxidant availability and the kinetics of metal recovery. From a design perspective, the measured K_L_a values are characteristic of a lab-scale stirred fine-bubble system and are lower than those typically reported for optimized industrial reactors such as venturi injectors or pressurized diffusion tanks. The overall ozone utilization efficiency, estimated from the difference between ozone input and residual ozone captured in the KI off-gas trap, was approximately 44.16% under the representative operating condition evaluated. This moderate value reflects limited gas–liquid contact time, incomplete ozone dissolution, off-gas losses, and ozone decomposition under near-neutral reactive conditions. Accordingly, scale-up strategies such as venturi injectors, fine-bubble or nanobubble-generating systems, and improved reactor hydrodynamics are expected to enhance ozone utilization and precipitation efficiency by increasing interfacial area, bubble stability, and residence time. These observations provide a basis for translating the laboratory results into scalable ozone-based metal recovery systems. Within this framework, the two-film theory remains useful for describing ozone transfer from the gas phase to the liquid phase, followed by reaction with dissolved metal species.^16,21,22,27^ ^,^ ^28,33,37,38^ ^,^ ^41,53,57^ For industrial recovery of Co, Mn, and related transition metals, process variables such as reactor configuration, gas flow rate, and agitation intensity should be systematically optimized to maximize oxidant utilization and precipitation efficiency (Figure 15). Overall, these findings provide a framework for understanding how operating variables collectively influence Co–Mn precipitation and for guiding subsequent process optimization.

Using controlled Co–Mn solutions enabled clear interpretation of the mass-transfer and precipitation behavior without interference from competing ions. These results offer a mechanistic basis for process optimization and facilitate translation of the approach to complex matrices such as acid mine drainage and industrial effluents. The Mn–Co precipitates generated in this study may serve either as mixed transition-metal oxide materials or as intermediate feedstocks for downstream hydrometallurgical purification using established separation techniques.

### 3.3 Economic and Environmental Considerations

Economic feasibility is an important consideration for evaluating the practical implementation of ozone-based oxidative precipitation and comparing it with conventional precipitation approaches for metal-bearing aqueous streams. Compared to conventional treatment methods, wastewaters such as AMD must often be treated regardless of resource recovery considerations, as required under the Clean Water Act (33 U.S.C. §1251). Conventional treatments (e.g., caustic soda or lime neutralization) are primarily designed to neutralize AMD, generating bulky sludge that requires disposal. Mn removal with conventional methods often requires raising pH to ∼ 10, followed by reneutralization or, alternatively, use of costly oxidants. In contrast, the ozone-driven process operates at circumneutral pH and utilizes ozone, a chemical-free oxidant, to recover Co, Mn, and Ni. This reduces chemical consumption and sludge volume, while enabling the selective recovery of multiple critical elements. Although ozone generation incurs energy costs, the reduction in chemical inputs, recovery of valuable metals, and avoidance of sludge disposal offer significant cost-offset potential and sustainability benefits. To provide additional context for process applicability, a screening-level operating-cost and recovered-metal-value analysis is provided in the Supporting Information (Section S2.3). This analysis places the measured direct electricity demand for ozone generation in the context of reported AMD treatment costs and the estimated gross contained value of recoverable Co and Mn for a representative AMD stream.

The transition from lab-scale to industrial-scale presents challenges related to gas distribution, bubble residence time, and reactor hydrodynamics. These factors are being addressed in our ongoing continuous and pilot-scale studies (∼10,000 GPD) to optimize reactor design, gas–liquid mass transfer efficiency, and process control. Kinetic data from this study will also support engineering design and economic modeling for large-scale implementation. To this end, a comprehensive life cycle assessment (LCA) and techno-economic analysis (TEA) will be investigated in future work.

Overall, this study provides mechanistic and practical insight into ozone-driven oxidative precipitation of Co and Mn from aqueous systems. The effects of gas flow rate, temperature, stirring rate, and initial ion concentration showed that Co recovery was strongly governed by ozone availability and gas–liquid mass transfer, whereas Mn precipitation remained favorable across a broader range of conditions because sufficient ORP was maintained for rapid oxidation. The two-film analysis and kinetic modeling together demonstrated that the process is primarily controlled by ozone transport and oxidant stability rather than intrinsic reaction kinetics, with K_L_a increasing from 0.0032 s^–1^ at 20 °C to 0.0057 s^–1^ at 40 °C and reaching 0.0147 s^–1^ at 1400 cc/min before declining at more severe conditions. In mixed systems, Mn oxide formation enhanced Co recovery through adsorption and coprecipitation, particularly at elevated temperature. Characterization by SEM–EDS, XRD, FT–IR, and XPS confirmed the formation of Co and Mn oxide phases and supported the role of Mn oxides in facilitating Co removal. Together, these results highlight the coupled roles of ozone mass transfer, precipitation kinetics, and nucleation behavior, providing a mechanistic foundation for optimizing and scaling ozone-based technologies for transition-metal recovery from aqueous resources.

## Supplementary Material



## Data Availability

All data supporting the findings of this study are presented within the article as figures and tables. No additional data sets were generated or deposited in external repositories.

## Notes

Detailed theoretical background on ozone properties and generation, two-film theory, chemical precipitation, and kinetics of precipitation (S1); Ozone properties (Table S1); The oxidation potential of various oxidizing agents (Figure S1); Results and Discussion (S2); Aqueous speciation and Eh–pH (Pourbaix) diagrams for Co and Mn (Figures S2–S3); Solubility of ozone in water as a function of temperature (Figure S4); Co–Mn Precipitation under optimal conditions in mixed solution (Figure S5); The activation energy for Co and Mn from three kinetic models (Figures S6 and S7); Solution Chemistry Study (S2.1), Box–Behnken parametric study for Co and Mn recovery (S2.2), and Economic and Environmental Considerations (S2.3) (PDF)
