# Supplementary material for: Chemical Kinetic and Mechanistic Investigation of Cobalt and Manganese Recovery from Aqueous Solutions by Ozone Oxidative Precipitation
Source: Environ Sci Technol. 2026 Jun 3;60(23):16928–43. doi: 10.1021/acs.est.5c18743 (PMC13276902; doi:10.1021/acs.est.5c18743)
Supplement: Supplementary file 1 [file es5c18743_si_001.pdf]

Supplementary Information (SI):

Chemical Kinetic and Mechanistic Investigation of Cobalt and Manganese Recovery from Aqueous Solutions by Ozone Oxidative Precipitation

Younes Shekarian; Mohammad Rezaee*; Sarma V. Pisupati

John and Willie Leone Family Department of Energy and Mineral Engineering, Center for Critical Minerals, College of Earth and Mineral Science, The Pennsylvania State University, University Park, Pennsylvania 16802, USA

* Corresponding author: m.rezaee@psu.edu

Manuscript Published in Environmental Science & Technology Journal

The Supplementary Information includes 2 sections, 15 pages, 7 figures, and 1 table.

SECTION S1. THEORETICAL BACKGROUNDS

SECTION S1.1 OZONE PROPERTIES AND GENERATION

Ozone, a highly reactive and unstable form of oxygen, has been extensively used in water treatment since the late 19th century. It appears as a blue gas with a distinct pungent smell.[1–3] Table S1 summarizes key properties of ozone relevant to its application in aqueous solutions.

Table S**1**: Ozone properties (adapted from[1–4])

**Property**	**Value**
Molecular weight	48 Da
Dipole moment	0.537 Debye
Density	2.14 Kg/m^3
Bond length	1.28 A
Oxidation potential	-2.07 V
Bond angle	117°
Melting point	–192.7°C
Boiling point	–110.5°C
Solubility in water at 0 °C	2.2 × 10^{-2} M
Solubility in water at 20 °C	1.19 × 10^{-2} M
Henry constant at 0 °C	35 atm M^{-1}
Henry constant at 20 °C	100 atm M^{-1}
Explosion threshold	10% Ozone

Due to its strong reactivity, ozone exhibits faster reaction rates with many contaminants compared to other oxidants, enabling shorter contact times and more efficient treatment. Moreover, ozone decomposes to pure oxygen without generating harmful residues, making it an environmentally friendly treatment agent. Because of its short half-life and inherent instability, ozone must be generated on-site, typically using ozone generators. This characteristic eliminates the need for handling, storage, or disposal of hazardous oxidants. Its rapid reaction kinetics and clean decomposition make it ideal for applications requiring fast and effective treatment without producing secondary pollution.[1,3–9] These advantages have led to widespread use of ozone across various industries. In water treatment, ozone is widely applied for drinking water and municipal wastewater.[1,3,10] In drinking water systems, ozone is used for bacterial disinfection, viral inactivation, oxidation of metals (e.g., iron, nickel, cobalt, and manganese), color and odor removal, and degradation of organic and inorganic pollutants, such as phenols, pesticides, cyanide, and sulfide. In municipal wastewater treatment, ozone contributes to the reduction of biological oxygen demand (BOD) and chemical oxygen demand (COD), oxidation of ammonia, and removal of suspended solids and micropollutants that are resistant to conventional treatment methods.[5] It also enhances microflocculation and serves as a pretreatment for biological processes. Emerging technologies such as ozone microbubbles and advanced oxidation processes (AOPs) have further improved ozone efficiency and pollutant degradation, particularly in high-strength industrial wastewater.[3] Beyond water treatment, ozone finds applications in aquaculture for pathogen control, in the food industry for sanitization, in healthcare for equipment sterilization, and in textiles for bleaching and effluent treatment, highlighting its versatility across sectors.[11,12]

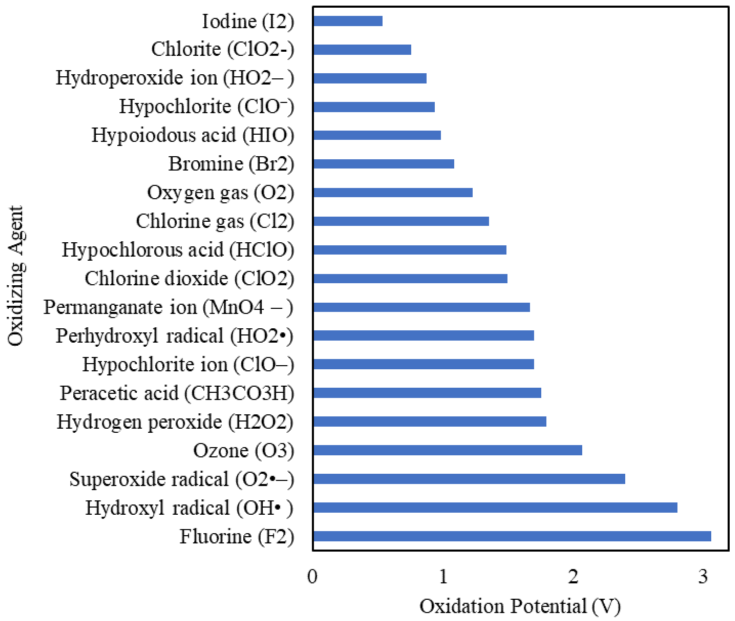

Figure S**1**: The oxidation potential of various oxidizing agents. [12–14]

Ozone is generated industrially via two primary methods: ultraviolet (UV) photolysis and electrical (corona) discharge. Both processes dissociate molecular oxygen (O_2) to form ozone (O_3) through subsequent recombination.

Section S1.1.1 UV LIGHT METHOD

UV-based ozone generation involves irradiating oxygen molecules with UV light at typically around 185 nm wavelength, as shown in RS1 and RS2:

O_2 + UV light (185 nm) → 2O (oxygen atoms) RS1

$O + O_2 \rightarrow O_3$ (ozone) RS2

The dissociated oxygen atoms combine with O_2 molecules to form ozone (RS2). Although the design and configuration of UV ozone generators might differ, they all operate on the fundamental principle of using UV radiation to trigger the reactions resulting in ozone formation. [8]

SECTION S1.1.2 ELECTRICAL DISCHARGE (CORONA) METHOD

Ozone can be produced using electrical or corona discharge techniques, which ionize oxygen molecules (O_2), enabling them to react and form ozone (O_3). When conditions are optimized, the bulk of the electron energy obtained in the electric field results in excited atomic and molecular states of oxygen (when O_2 and air are the feed gases) and nitrogen (when air is the feed gas). The excited states of O_2 (O_2*) then dissociate according to reactions RS3 and RS4.[1,7]

$O_2 + e- \rightarrow O_2^* + e^-$ RS3

$O_2^* \rightarrow 2O$ RS4

$O + O_2 + M \rightarrow O_3^* + M \rightarrow O_3 + M$ RS5

Where *M* represents a collision partner such as nitrogen or oxygen, and O_3* represents the initial transient excited state of ozone. The ozone generation in reaction RS5 competes with reactions RS6-RS8, which also use oxygen atoms.[15]

$O + O + M \rightarrow O_2 + M$ RS6

$O + O_3 + M \rightarrow 2O_2 + M$ RS7

$O + O_3^* + M \rightarrow 2O_2 + M$ RS8

When air is used as the feed gas, several nitrogen species, such as N^+, N^{2+}, and N and excited atomic and molecular states increase the complexity of the reaction system. This results in additional reactions RS9-RS11, involving nitrogen atoms and excited molecular states of N_2.

$N + O_2 \rightarrow {}^{\bullet}NO + O$ RS9

$N + {}^{\bullet}NO \rightarrow N_2 + O$ RS10

$N_2 + O_2 \rightarrow N_2O + 2O$ RS11

Roughly 50% of the ozone produced in air-fed systems arises from these nitrogen-based processes. Ozone formation through these methods is slower (approximately 100 μs) than in O_2 (10 μs). Furthermore, several other nitrogen oxide species like ${}^{\bullet}NO_2$, ${}^{\bullet}NO_3$, and N_2O_5 are formed, which consume ozone (RS12-RS15).[1,7]

${}^{\bullet}NO + O_3 \rightarrow {}^{\bullet}NO_2^* + O_2$ RS12

${}^{\bullet}NO_2^* \rightarrow {}^{\bullet}NO_2 + h\upsilon$ RS13

$2{}^{\bullet}NO + 3O_3 \rightarrow N_2O_5 + 3O_2$ RS14

${}^{\bullet}NO_2 + O_3 \rightarrow {}^{\bullet}NO_3 + O_2$ RS15

Moreover, moisture in the feed gas can interfere with the ozone generation process and produce undesired byproducts. When moisture is present in the feed gas during ozone generation, the singlet O atom, $O(^1D)$, reacts very quickly with water vapor, and it can react with ozone to form various undesired byproducts, such as hydrogen peroxide (H_2O_2) and nitric acid (HNO_3) (RS16-RS19).

$O(^1D) + H_2O \rightarrow H_2O_2^* \rightarrow 2\ {}^{\bullet}OH$ RS16-17

$\bullet OH + \bullet NO \rightarrow HNO_2$ RS18

$\bullet OH + \bullet NO_2 \rightarrow HNO_3$ RS19

Therefore, the formation of nitrous acid (HNO_2) and nitric acid (HNO_3) can occur during the ozone generation process, particularly when the air feed is not dried.[1]

SECTION S1.2 CHEMICAL REACTION (PRECIPITATION)

Once dissolved in water, ozone undergoes irreversible oxidation reactions with the transition metal ions, converting soluble species into insoluble oxides or oxyhydroxides that readily precipitate. For Co and Mn, the overall reactions are as follows:

$2Co^{2+} + O_3 + 3H_2O \rightarrow 2CoOOH + 4H^+ + O_2$ $\Delta G^0 = -61.76$ kJ/mol RS20

$Mn^{2+} + O_3 + H_2O \rightarrow MnO_2 + 2H^+ + O_2$ $\Delta G^0 = -164.05$ kJ/mol RS21

The precipitation process entails the formation of a solid phase within a saturated solution, involving several overlapping subprocesses: crystal nucleation, growth, Ostwald ripening, and agglomeration. Nonetheless, in practice, these subprocesses are often not entirely distinguishable.[16] At high supersaturation, homogeneous and heterogeneous nucleation dominate, yielding fine particles (0.1–10 μm) with high surface energy. These conditions promote secondary processes that affect particle morphology and settling behavior. Supersaturation serves as the principal driving force for precipitation, directly influencing the mechanistic pathways and kinetics of nucleation, crystal growth, and ripening. Thus, a thorough understanding of both thermodynamics and kinetics is essential to effectively control the precipitation process and enhance selectivity and recovery.[17]

SECTION S1.3 DOUBLE-FILM THEORY FOR GAS-LIQUID MASS TRANSFER

SECTION S1.3.1 THEORETICAL CALCULATION OF K_La

The theoretical volumetric mass transfer coefficient, K_La, quantifies the rate at which ozone dissolves into water and is estimated using the dimensionless Sherwood number (Sh):

$$Sh = 2 + 0.6Re^{0.50}Sc^{0.33} \qquad \textit{Eq.(S1)}$$

In this equation, Re denotes the Reynolds number and is expressed as $Re = \frac{U_g d_b}{\nu}$, where U_g denotes the superficial gas velocity, d_b refers to the bubble diameter, and ν corresponds to the kinematic viscosity of the liquid. The Schmidt number (Sc) characterizes the relative effectiveness of momentum versus mass transport and is given by $Sc = \frac{\nu}{D}$, where D represents the ozone diffusivity in liquid.

The individual mass transfer coefficient K_L signifies the rate at which ozone transfers across the liquid boundary layer and is calculated as:

$$K_L = \frac{Sh.D}{d_b} \qquad \textit{Eq.(S2)}$$

The overall volumetric mass transfer coefficient is then determined using:

$$K_L\text{a} = K_L * \text{a} \qquad \text{E}\textit{q.(S3)}$$

where a designates the specific interfacial area between the gas and liquid phases, estimated based on sparger geometry, gas holdup, and bubble size.

SECTION S1.3.2 EXPERIMENTAL DETERMINATION OF K_La

Experimentally, the volumetric mass transfer coefficient K_La was determined using the iodometric method, which measures the concentration of undissolved ozone via its reaction with potassium iodide (KI) solution. The total amount of ozone introduced into the system was obtained from the calibrated output of the ozone generator (e.g., 16.66 mg/min at 200 cc/min O_2 flow rate), and the ozone remaining in the gas phase was quantified by directing the effluent gas stream through a KI solution trap and performing titration. The amount of ozone absorbed into the liquid phase was then calculated by subtracting the undissolved ozone from the total amount generated. The mass transfer coefficient was then calculated using the integrated form of the first-order, pseudo-steady-state mass transfer equation:

$$K_L\text{a} = -\frac{1}{\text{t}} \ \ln\left(1 - \frac{C}{C^*}\right) \qquad \textit{Eq.(S4)}$$

Where C denotes the measured ozone concentration in solution at time t, and C^* signifies the equilibrium concentration of ozone in the liquid, governed by Henry's law.

The equilibrium concentration, C^*, was calculated using Henry's law, which relates the gas-phase partial pressure of ozone to its solubility in water, and is expressed as:

$$C^* = \text{H} * P_{O_3} \qquad \textit{Eq.(S5)}$$

Here, H represents the Henry's law constant (mg/L·atm), which is temperature-dependent, and P_{O_3} signifies the partial pressure of ozone in the gas stream (atm).

To quantify the amount of ozone dissolved in the liquid phase, the experimental setup directed the gas stream exiting the reactor into an absorber containing a potassium iodide (KI) solution.[18,19] Unreacted ozone reacted with iodide to produce molecular iodine (I_2) according to the following reaction:

$O_3 + 2KI + H_2O \rightarrow 2KOH + O_2 + I_2$ RS22

The amount of iodine formed was then quantified by titrating the KI absorber solution with a standardized sodium thiosulfate ($Na_2S_2O_3$) solution. The volume of titrant used directly corresponded to the moles of iodine (and therefore ozone) present, and by extension, the ozone that had reacted. By subtracting the amount of ozone recovered in the absorber (as calculated from titration) from the total ozone injected, the actual ozone uptake by the liquid phase was determined. This indirect but reliable measurement served as the basis for determining the volumetric mass transfer coefficient K_La as described in Section 2.1.1.

SECTION S1.4 KINETICS OF PRECIPITATION

The Pseudohomogeneous model is a more complex model that attempts to bridge the Linear and Higbie models.[20,21] It considers both the reaction kinetics (like the Linear model) and mass transfer limitations (like the Higbie model). The rate equation is as follows:

$\ln(C_t/C_o) = -k'' * t$ *Eq.(S6)*

Where k'' is a precipitation rate, C_t and C_o are elemental concentrations (e.g., Co and Mn) at time t from the beginning of the reaction, respectively. Considering both reaction kinetics and mass transfer, the Pseudohomogeneous model can provide a more comprehensive understanding of the system's behavior. However, this model is more complex, requiring more information (such as the reaction rate constant and mass transfer coefficient) and may involve more challenging computational analysis. In this model, the resistance to mass transfer from the gas phase to the liquid phase was found to be negligible, and the amount of ozone dissolved in the liquid was regarded as abundant. [20,21]

The activation energy (E_a) of the reaction was also calculated through Arrhenius Eq.(S7):

$k = A\ exp(-E_a/RT)$ *Eq.(S7)*

Where A is the frequency at which atoms and molecules collide in a way that leads to a reaction, R is the gas constant, T represents the temperature in Kelvin, and k is the temperature-dependent constant.[16,22]

SECTION S2. RESULTS AND DISCUSSION

SECTION S2.1 SOLUTION CHEMISTRY STUDY

Saturation index (SI) was calculated to evaluate the potential for precipitation of Co-Mn hydroxides or oxides under specific conditions. The process of manganese(II) and Cobalt(II) being oxidized by ozone in water to form manganese(III) oxide and cobalt(III) oxide can be written as follows:

$Mn^{2+} + 0.5O_3 + H_2O = 0.5Mn_2O_3 + 2H^+ + 0.5O_2$ $\Delta G^0 = -470.49$ kJ/mol RS23

$Co^{2+} + O_3 + H_2O = 0.3Co_3O_4 + 2H^+ + 1.3O_2$ $\Delta G^0 = -136.51$ kJ/mol RS24

If SI > 0, the solution is supersaturated, and there is a potential for the precipitation of Mn_2O_3 and Co_3O_4. If SI = 0, the solution is at equilibrium. If SI < 0, the solution is undersaturated, meaning more Mn_2O_3 and Co_3O_4 can dissolve into the solution.

The previous study by the authors on the effects of various ligands/oxidants on Co-Mn showed that ozone was the most effective oxidizing agent for the recovery of these elements from AMD. To further explore how ozone can influence the precipitation of Co-Mn, the SI was calculated based on the concentration of these elements in the stock solutions (Fig. 3a). The results showed SI values for Co and Mn oxide formation are positive at pH around 2 in the presence of ozone, indicating favorable conditions for oxidative precipitation. In contrast, precipitation using hydroxide ligand required elevated pH values (> 9) to achieve positive SI values for $Co(OH)_2$ and $Mn(OH)_2$. Moreover, in all corresponding pH, the values of SI for $Co(OH)_2$ and $Mn(OH)_2$ were consistently lower than those for their oxide counterparts (i.e., Co_3O_4 and Mn_2O_3) due to the higher solubility of Mn-Co (II)-hydroxide in aqueous systems. These results indicate that ozone enables a more favorable pathway for metal precipitation, especially under mildly acidic or neutral conditions where traditional alkaline precipitation is ineffective.

Thermodynamic analysis further supports the preferential oxidative precipitation of Mn over Co, a trend confirmed in previous studies.[23]. This aligns with the redox potential required for their precipitation. As shown in Figs. 3b, the oxidative precipitation diagram at pH 7 confirms that both Co and Mn can be selectively precipitated using ozone, aligned with RS23-RS24. The solution chemistry data agree with our previous experimental results, as more than 95% of Co and Mn were recovered from AMD using ozone as an oxidizing agent at acidic or neutral pH.[24]

Fig. S2 illustrates the aqueous speciation diagrams for Mn and Co as a function of pH. For Mn, the dominant species in acidic solutions (pH < 5) is soluble Mn^{2+}, which transitions to MnO_2(s) between pH 5 and 10. At even higher pH (>12), the formation of soluble permanganate (MnO_4^-) becomes thermodynamically favorable (Fig. S2a). In contrast, Co^{2+} remains the primary aqueous species up to pH ~7, above which it begins to precipitate as $Co(OH)_2$(s), followed by $Co(OH)_3$(s) at pH > 9 (Fig. S2b).

Eh-pH (Pourbaix) diagrams illustrated in Fig. S3 identify redox-pH stability zones and support ozone-based precipitation over a wide pH range. The results show that Co and Mn could precipitate via the oxidation pathway even from acidic and neutral solutions (e.g., MnO_2 or MnOOH formation through arrow A2 path in Fig. S3) to neutral pH through oxidative precipitation. Similarly, precipitation using alkaline ligands such as NaOH and NH_4OH is feasible near pH 10, forming $Mn(OH)_2$ and $Co(OH)_2$ via path A1 in Fig. S3. Overall, the SI and Pourbaix analyses confirm that ozone is a highly effective oxidizing agent for Co and Mn recovery from aqueous solutions, particularly under conditions where pH adjustment alone is insufficient.

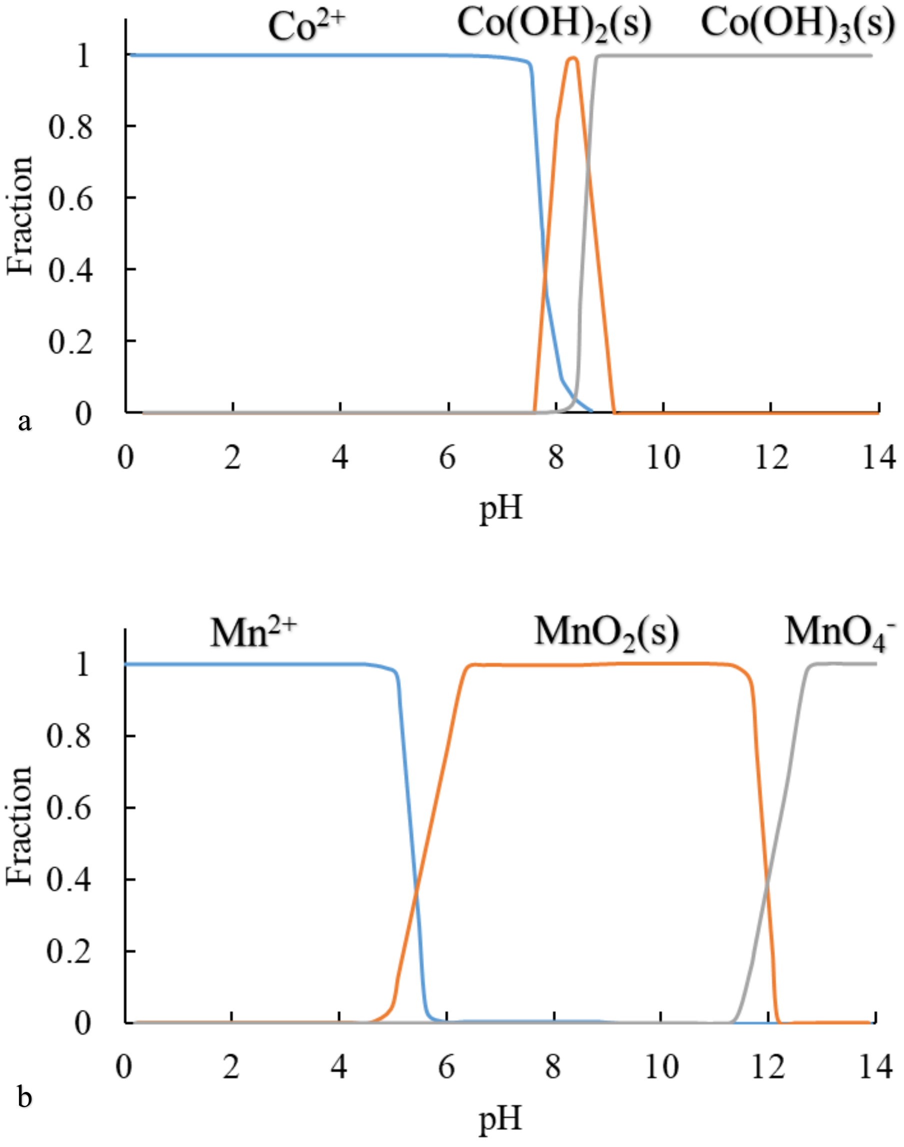

Figure **S2**: Speciation diagrams for both pure Co (a) and Mn (b) at equilibrium with varying pH in H_2O system at 25 °C.

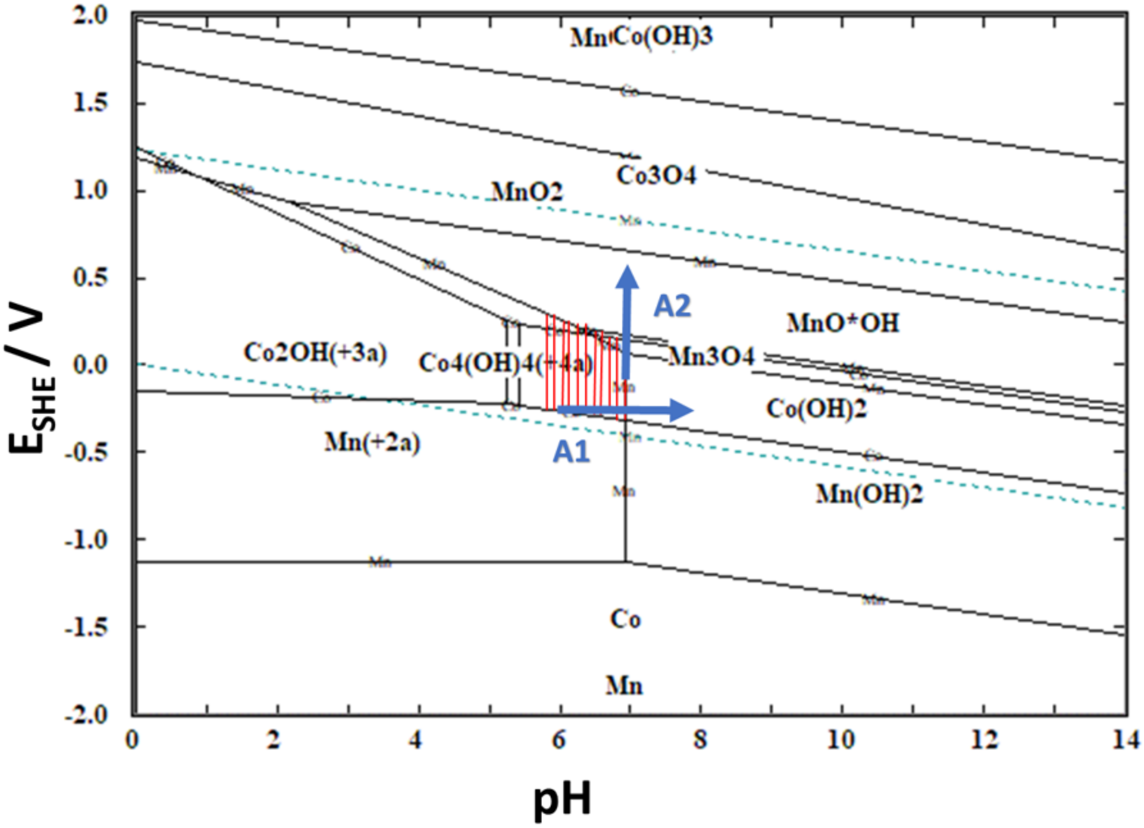

Figure **S3**: Eh-pH diagrams for Co and Mn showing the predominant species in H_2O system at 25 °C.

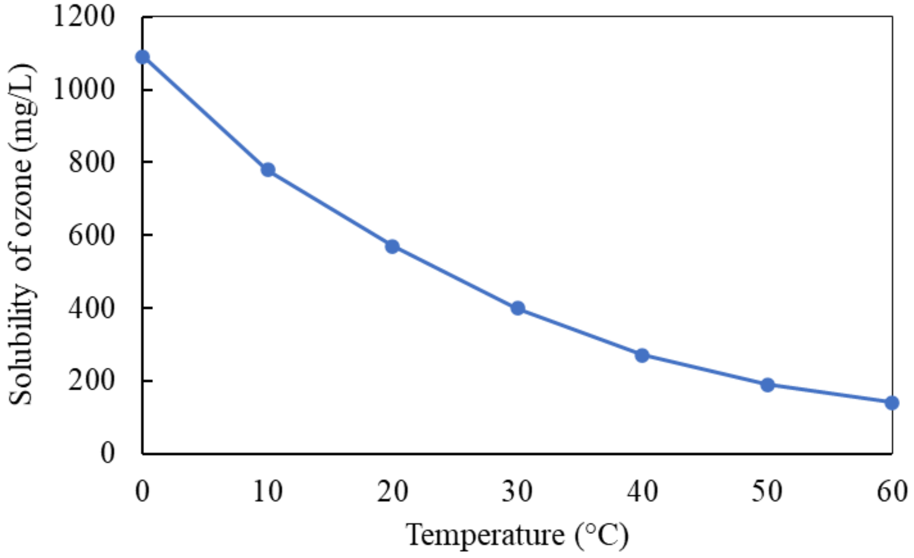

Figure **S4**: Solubility of ozone in water as a function of temperature (Data taken from[25–27]).

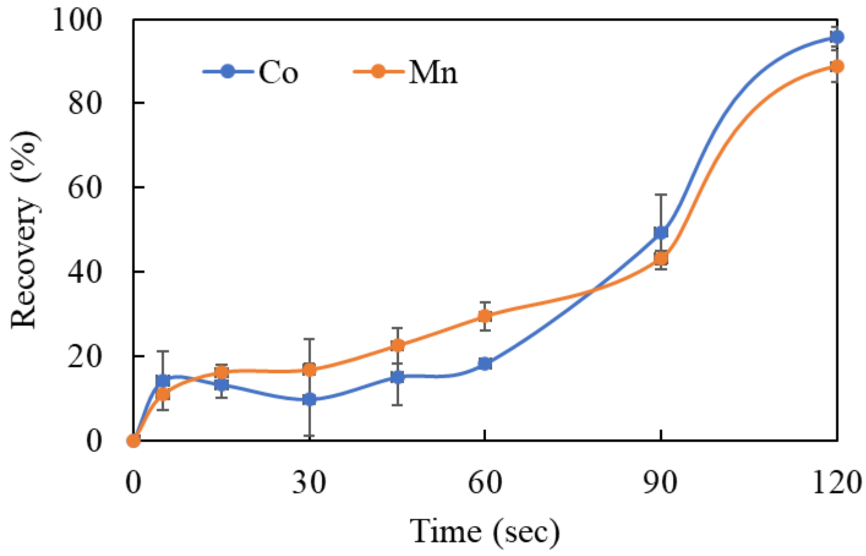

Figure **S5**: Co-Mn Precipitation under optimal conditions in mixed solution (T=80 ºC, flow rate: 1400cc/min, stirring rate=1500 rpm).

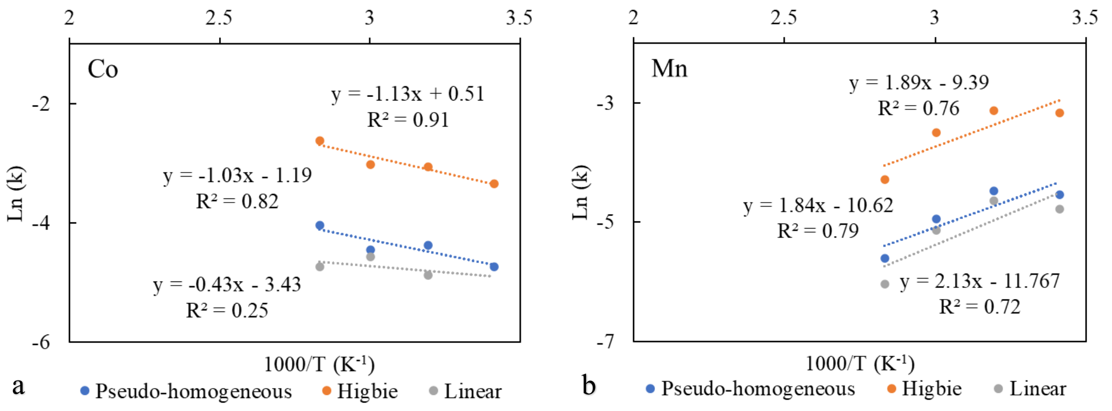

Figure **S6**: The activation energy for Co (a) and Mn (b) oxidative precipitation in pure solution

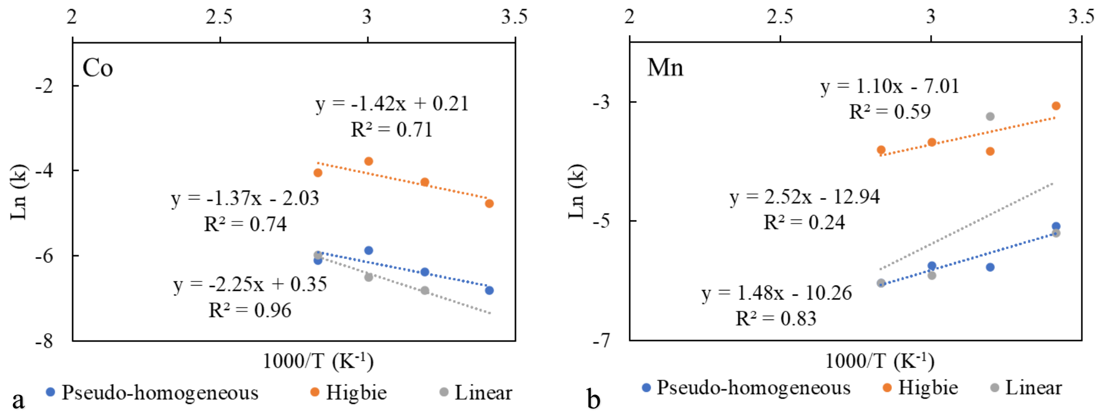

Figure **S7**: The activation energy for Co (a) and Mn (b) oxidative precipitation in mixed solution.

SECTION S2.2 PARAMETRIC EVALUATION

A statistically designed parametric study using a three-level Box-Behnken design was performed to evaluate the influence of key operating parameters and their interactive effect on the oxidative precipitation of Co-Mn from an aqueous solution. The parameters tested include gas flow rate, stirring rate, and temperature, each at three levels, as shown in Table 1 of the main manuscript. A total of 17 experiments, including five repeat tests at the center points, were conducted to assess experimental error and ensure reproducibility of the results. This approach, a form of response surface methodology (RSM), enables the efficient modeling of nonlinear relationships between multiple independent variables and response variables, while minimizing the number of experiments.[28,29] The experimental program evaluated the effects of flow rate, temperature, and stirring rate on the recovery of Co and Mn from pure solutions. Replicate runs were included to assess experimental error.

First, the statistical analysis applied one-way analysis of variance (ANOVA) to test for statistically significant differences among population means. In ANOVA, the null hypothesis assumes that the process parameters (temperature, stirring rate, and flow rate) have no significant effect on the oxidative precipitation of Co and Mn at 5% significance level.[30]

The ANOVA results for the Co recovery model yielded a statistically significant regression (p-value = 0.0013) and a non-significant lack of fit (p-value = 0.3190), indicating that the quadratic model adequately describes the observed data (Fig. 15a). Among three input parameters, temperature (p-value=0.0003) and flow rate (p-value=0.0136) were identified as significant factors on Co recovery. The surface response plot (Fig. 15a) showed that Co recovery initially increases with increasing temperature and flow rate, reaches a maximum, and then decreases. This trend is consistent with earlier observations in Fig. 7, where higher temperatures enhance reaction kinetics up to a point but also promote ozone decomposition, reducing oxidant availability. The optimum condition for Co recovery was found to be a flow rate of 1100 cc/min, and a temperature of 50 ^{o}C.

For Mn recovery, the ANOVA results also indicated a statistically significant model (p = 0.0196) and a non-significant lack of fit (p = 0.6348) (Fig. 15b). The main effect of flow rate and its interactive effects with both temperature (p=0.0113) and stirring rate (p=0.0328) were statistically significant. The surface response plot (Fig. 15b) revealed that Mn recovery increases with both temperature and flow rate, with no decline at higher levels, unlike Co. This pattern corroborates previous experimental findings (see Fig. 7) showing that Mn precipitation is less affected by ozone decomposition and more thermodynamically favored at elevated temperatures. The optimum condition for Mn recovery was obtained at a flow rate of 2000 cc/min and a temperature of 80 ^{o}C.

The use of model Co–Mn solutions in this study allowed precise evaluation of the kinetic and mechanistic pathways governing ozone oxidative precipitation under near-neutral conditions. This controlled approach provided clear insight into the intrinsic reaction behavior of Co and Mn without interference from complexing agents or competing ions, establishing a reliable foundation for subsequent application to real effluents. In more complex systems such as AMD or industrial wastewater, where acidity and ionic strength are higher, the insights gained here will facilitate process optimization and the selective separation and recovery of Co and Mn from multi-component aqueous matrices, contributing to the development of sustainable hydrometallurgical processes.

SECTION S2.3 ECONOMIC AND ENVIRONMENTAL CONSIDERATIONS

To place the cost of ozone treatment in context, reported costs for active abandoned mine drainage treatment facilities operated by the Pennsylvania Department of Environmental Protection Bureau of Abandoned Mine Reclamation (PA DEP-BAMR), primarily associated with legacy coal mine drainage, range from \$0.008 to \$0.637 per 1000 gal treated, with an average of \$0.234 per 1000 gal.[31] These values indicate that AMD treatment is already a recurring operating cost even when no resource recovery is included. Therefore, the economic relevance of ozone oxidative precipitation should be evaluated not only in terms of direct treatment cost, but also in terms of its dual function as a water-treatment oxidant and a critical-mineral recovery agent. Ozone is widely used in water and wastewater treatment for oxidation of dissolved metals, degradation of organic contaminants, disinfection, and odor control. In the present application, ozone provides an additional benefit by enabling selective Co-Mn recovery at circumneutral pH, thereby integrating contaminant removal with resource recovery. To quantify the main operating cost associated with ozone oxidative treatment, the electricity consumption of the ozone generator was measured under the optimized recovery conditions and expressed per unit volume of treated solution. Because near-complete Co-Mn recovery was achieved within approximately 2 min (Fig. S5), the measured direct ozone-generation electricity demand was calculated as 1.6 kWh/m^3 of treated solution for the 500 mL batch volume used in this study. Based on U.S. Energy Information Administration electricity price data for February 2026, the average U.S. industrial electricity rate was 8.95 cents kWh^{-1}, equivalent to \$0.0895 kWh^{-1}.[32] Using this industrial rate, the direct electricity cost associated with ozone generation is approximately \$0.14 m^{-3}, or \$0.54 per 1000 gal.

To place this operating energy cost in the context of recoverable critical-mineral value, a representative AMD stream with an average flow rate of 100 gpm was considered. This flow rate corresponds to 144,000 gal per day. For an AMD stream containing 10 ppm Co and 50 ppm Mn, with 99% recovery for both metals, the process would produce approximately 5.40 kg Co per day and 26.98 kg Mn per day. Assuming continuous operation for 365 days per year, this corresponds to approximately 1,971 kg Co per year and 9,854 kg Mn per year. Using approximate market values of \$56.29 per kg for Co[33] and \$1.31 per kg for Mn[34], the gross contained Co-Mn value is approximately \$0.628 m^{-3} or \$2.38 per 1000 gal of AMD treated. At the same flow rate, this corresponds to approximately \$339 per day, or \$124,000 per year in gross contained critical-mineral value.

This comparison provides a screening-level indication that the value of recoverable Co and Mn in AMD can be meaningful relative to the direct electricity demand for ozone generation, particularly when treatment is already required for environmental compliance. The potential value of the ozone approach lies in combining AMD treatment with selective critical-mineral recovery at circumneutral pH, reducing chemical addition and secondary salt loading, decreasing sludge liability, and producing a Co-Mn-rich precipitate that may serve as an intermediate feedstock for downstream hydrometallurgical purification.

REFERENCES

1. Von Sonntag, C.; Von Gunten, U. Chemistry of Ozone in Water and Wastewater Treatment : From Basic Principles to Applications. *IWA Publishing*. **2012**.
2. Xiao, J.; Xie, Y.; Cao, H. Organic pollutants removal in wastewater by heterogeneous photocatalytic ozonation. *Chemosphere,* **2015**. 121, 1-17.
3. Rekhate, C. V.; Srivastava, J. K. Recent advances in ozone-based advanced oxidation processes for treatment of wastewater- A review. *Chemical Engineering Journal Advances.* **2020**, 3, 100031.
4. Varga, L.; Szigeti, J. Use of ozone in the dairy industry: A review. *International Journal of Dairy Technology.* **2016**, 69(2), 157–168.
5. Alsheyab, M. A. T.; Muñoz, A. H. Optimisation of ozone production for water and wastewater treatment. *Desalination.* **2007**, 217, 1–7.
6. Dodd M. C. Characterization of ozone-based oxidative treatment as a means of eliminating target-specific biological activities of municipal wastewater-borne antibacterial compounds. *ETH Zurich.* **2008**.
7. Kogelschatz, U.; Baessler, P. Determination of Nitrous Oxide and Dinitrogen Pentoxide Concentrations in the Output of Air-Fed Ozone Generators of High Power Density. *Ozone Sci. Eng.* **1987**, 9, 195–206.
8. McCollum, B. S. Mastering the Fundamentals of Ozone- Ozone Generation. *Water Quality Product, the IOA*. **2008**, 90809.
9. Tian, Q.; Xin, Y.; Wang, H.; Guo, X. Potential-controlled selective recovery of manganese and cobalt from cobalt slag leaching solution. *Hydrometallurgy.* **2017**, 169, 201–206.
10. Gottschalk, C.; Libra, J. A.; Saupe, A. *Ozonation of Water and Waste Water*. *Wiley.* **2009**, doi:10.1002/9783527628926.
11. Guzel-Seydim, Z. B.; Greene, A. K.; Seydim, A. C. Use of ozone in the food industry. *LWT - Food Science and Technology.* **2004**, 37, 453–460.
12. Epelle, E. I.; Macfarlane, A.; Cusack, M.; Burns, A.; Okolie, J. A.; Mackay, W.; Rateb, M.; Yaseen, M. Ozone application in different industries: A review of recent developments. *Chemical Engineering Journal.* **2023**,454, 140188.
13. Coyle, E. E.; Ormsbee, L. E.; Brion, G. M. Peracetic Acid as an Alternative Disinfection Technology for Wet Weather Flows. *Water Environment Research.* **2014**, 86, 687–697.
14. Guzel-Seydim, Z. B.; Greene, A. K.; Seydim, A. C. Use of ozone in the food industry. *LWT - Food Science and Technology.* **2004**, 37, 453–460.
15. Kogelschatz, U. Dielectric-barrier discharges: their history, discharge physics, and industrial applications. *Plasma Chemistry and Plasma Processing.* **2003**, 23, 1–46.
16. Vaziri Hassas, B.; Shekarian, Y.; Rezaee, M. Selective precipitation of rare earth and critical elements from acid mine drainage - Part I: Kinetics and thermodynamics of staged precipitation process. *Resour. Conserv. Recycl.* **2023**, 188.
17. Myerson, A. Handbook of IndustriaI CrystalIization Second Edition. *Butterworth-Heinemann*. **2002**.
18. Ratnawati, R.; Kusumaningtyas, D. A.; Suseno, P.; Prasetyaningrum, A. Mass Transfer Coefficient of Ozone in a Bubble Column. *MATEC Web of Conferences.* **2018**, 156, 02015.
19. Rodriguez-Blanco, J. D.; Vallina, B.; Blanco, J. A.; Benning, L. G. The role of REE $^{3+}$ in the crystallization of lanthanites. *Mineral. Mag.* **2014**, 78, 1373–1380.
20. Cruz-Díaz, M. R.; Arauz-Torres, Y.; Caballero, F.; Lapidus, G. T.; González, I. Recovery of MnO2 from a spent alkaline battery leach solution via ozone treatment. *J. Power Sources.* **2015**, 274, 839–845.

21. Oruê, B. P.; Botelho Junior, A. B.; Tenório, J. A. S.; Espinosa, D. C. R.; Baltazar, M. D. P. G. Kinetic Study of Manganese Precipitation of Nickel Laterite Leach Based-solution by Ozone Oxidation. *Ozone Sci. Eng.* **2021**, 43, 324–338.
22. Lewis, A.; Seckler, M.; Kramer, H.; Rosmalen, G. V. *Industrial Crystallization: Fundamentals and Applications*. **2015**.
23. Ichlas, Z. T.; Mubarok, M. Z.; Magnalita, A.; Vaughan, J.; Sugiarto, A. T. Processing mixed nickel-cobalt hydroxide precipitate by sulfuric acid leaching followed by selective oxidative precipitation of cobalt and manganese. *Hydrometallurgy.* **2020**, 191.
24. Shekarian, Y.; Hassas, B.; Rezaee, M.; Pisupati, S. V. Development of a chemical-free process utilizing ozone oxidative precipitation for the recovery of cobalt and manganese from acid mine drainage. *J. Environ. Chem. Eng.* **2022**, 10, 108533.
25. Langlais, B.; Reckhow, D. A.; Brink, D. R. *Ozone in Water Treatment. Application and Engineering.* **1991**, 558.
26. Caprio, V.; Insola, A.; Lignola, P. G.; Volpicelli, G. A new attempt for the evaluation of the absorption constant of ozone in water. *Chem. Eng. Sci.* **1982**, 37, 122–124.
27. Ullmann, F. Encyclopedia of Industrial Chemistry. *Wiley*, Federal Republic of Germany. **1991**.
28. Box, G. E. P.; Draper, N. R. Review of Empirical Model-Building and Response Surfaces. *Contemporary Psychology: A Journal of Reviews.* **1989**, 34, 518–518.
29. Rezaee, M., Warner, R. C.; Honaker, R. Q. Development of an electrical conductivity screening test for mine waste assessments. *Chemosphere.* **2016**, 160, 13–21.
30. Moore, D. S.; McCabe, G. P. Introduction to the Practice of Statistics. *New York: WH Freeman*. **2009**, 4.
31. Beam, R. L. Overview of Acitive Mine Drainage Treatment Facilities Currently Operated by the PA-DEP Bureau of Abandoned Mine Reclamation. *West Virginia Mine Drainage Task Force Symposium, Morgantown, WV*. **2019**.
32. U.S. Energy Information Administration. Electricity Monthly Update: End Use, February 2026; *Washington*, *DC*, 2026. Accessed May 3, **2026**.
33. Trading Economics. Cobalt Price Chart - Historical Data - Accessed May 4, **2026**.
34. IMARC Group. Manganese Prices, Trend, Chart, Demand, Market Analysis, News, Historical and Forecast Data Report 2026 Edition. SR112026A23410, Accessed May 4, **2026**.
